# Immune Cell Hacking: Challenges and Clinical Approaches to Create Smarter Generations of Chimeric Antigen Receptor T Cells

**DOI:** 10.3389/fimmu.2018.01717

**Published:** 2018-07-31

**Authors:** Reza Elahi, Elnaz Khosh, Safa Tahmasebi, Abdolreza Esmaeilzadeh

**Affiliations:** ^1^School of Medicine, Zanjan University of Medical Sciences, Zanjan, Iran; ^2^Department of Immunology, Health Faculty, Tehran University of Medical Sciences, Tehran, Iran; ^3^Department of Immunology, Zanjan University of Medical Sciences, Zanjan, Iran; ^4^Cancer Gene Therapy Research Center (CGRC), Zanjan University of Medical Sciences, Zanjan, Iran

**Keywords:** adoptive cell therapy, chimeric antigen receptor T cell therapy, immunotherapy, clinical applications, immune cell hacking, challenges

## Abstract

T cells equipped with chimeric antigen receptors (CAR T cells) have recently provided promising advances as a novel immunotherapeutic approach for cancer treatment. CAR T cell therapy has shown stunning results especially in B-cell malignancies; however, it has shown less success against solid tumors, which is more supposed to be related to the specific characteristics of the tumor microenvironment. In this review, we discuss the structure of the CAR, current clinical advantages from finished and ongoing trials, adverse effects, challenges and controversies, new engineering methods of CAR, and clinical considerations that are associated with CAR T cell therapy both in hematological malignancies and solid tumors. Also, we provide a comprehensive description of recently introduced modifications for designing smarter models of CAR T cells. Specific hurdles and problems that limit the optimal function of CAR T cells, especially on solid tumors, and possible solutions according to new modifications and generations of CAR T cells have been introduced here. We also provide information of the future directions on how to enhance engineering the next smarter generations of CAR T cells in order to decrease the adverse effects and increase the potency and efficacy of CAR T cells against cancer.

## Introduction

To date, cancer treatment included chemotherapy, radiotherapy, and surgery. According to multiple side effects, low efficacy, and high risk of relapse accompanied by prior treatment methods, novel treatment strategies with higher efficacy and lower side effects have been introduced (1). Immunotherapy, boosting patient’s own immune system to fight diseases, has recently attracted much attention as a new treatment method for cancer. Adoptive cell therapy (ACT) is among the latest progressions in immunotherapy (2–4). One encouraging method of the ACT is the adoptive transfer of genetically engineered T cells to express chimeric antigen receptor (CAR). Eshar recommended the initial theory of CAR in 1989, which proposed to equip T cell with CAR in order to redirect it against a specific tumor antigen (5–8).

Chimeric antigen receptor is a kind of engineered T cell receptor with the ability to recognize a pre-defined target Ag and introduce it to the T cell, in order to activate its cytotoxicity against target cells. CAR T cell recognizes tumor surface antigen in an antibody-like recognition pattern which is independent of MHC (9). This enables CAR T cells to recognize an extensive range of targets including proteins, carbohydrates, and glycopeptides. After distinguishing the specific target antigen on tumor cells, CAR T cells would be able to kill them (10).

Chimeric antigen receptor is composed of four domains including extracellular domain, hinge or spacer, transmembrane (TM), and intracellular domain. The extracellular domain is typically constructed from the Single chain Variable Fragment (ScFV) part of a specific antibody which is directed against the target antigen. The Spacer/Hinge domain is usually made of IgG_1_ and influences the flexibility of extracellular domain and function of the CAR T cell. TM domain is mostly derived from CD8/CD28 and affects the expression of CAR on T cell membrane. The intracellular domain consists of CD3 signaling pathway which activates the T cell after binding to the target cell. Co-stimulatory domains such as CD28 and 4-1BB, which are applied for construction of second and third generation of CAR T cells, can improve the proliferation, cytokine production, anti-tumor potency, and persistence of the T cell by providing the secondary signaling pathway (11).

**Figure 1:**
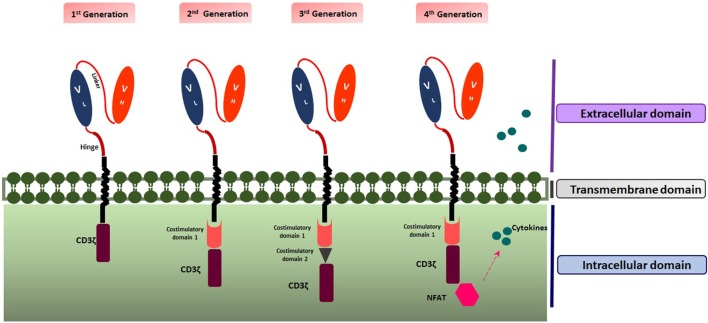
Structure of the four generations of chimeric antigen receptors. Created by Esmaeilzadeh et al.

Up to now, four generations of CAR T cells have been introduced. The first generation (1G) includes ScFV as the target recognition and CD3ζ signaling chain as the intracellular domain. The second generation (2G) encompasses a co-stimulatory domain such as 4-1BB (CD137) or CD28 as the secondary signal producer in addition to properties of the first generation. Applying both co-stimulatory domains including CD28 and 4-1BB led to the construction of the third generation (3G) of CAR T cells (12). Moreover, the fourth generation (4G) also named as T cell Redirected for Universal Cytokine Killing (TRUCK T cell) or armored CAR T cell, combines the properties of the 2G with enhanced ability to be more efficient against the tumor, such as the capability of cytokine secretion. Multiple cytokines such as interleukin-15 (IL-15) (13) and IL-12 have been recruited to empower CAR T cell therapy against the cancer cells (2) (Figure 1).

The process of producing CAR T cells for the clinical application includes multiple steps. First, mononuclear cells are isolated from patient’s peripheral blood. Then, stimulation/activation of T cells is performed *via* monoclonal antibodies (such as anti-CD28 and anti-CD3) or cytokines (such as IL-2, IL-15, and IL-17). After stimulation, the transgene encoding CAR is transfected to the T cell through viral or non-viral approaches such as retroviral and lentiviral vectors, transposon (including Sleeping Beauty and PiggyBac), and plasmid; however, most clinical trials have employed retroviral vectors for gene transfer (14). Special characteristics and limitations of each vector are addressed in Table 1.

**Table 1:** Characteristics and limitations of each vector utilized for chimeric antigen receptor (CAR) transgene transduction.

Vector	Special properties	Limitations
Gammaretroviral	Integration into the cell genome (15)	Insertional oncogenesis (15)
		High expense and cost (16)
	Permanent expression of the gene (16)	Affecting active dividing cells (15)
	Availability of multiple packaging systems (15)	Decrease in expression of CAR after a while (16)
		Restricted cargo capability (15)

Lentiviral	Affecting non-dividing cells	Missing extensive accessible vector packing systems (18)
	Improved cargo capability (17)	Diverse lot-to-lot features (17)
	Decreased chance of insertional oncogenesis (18)	

Transposon	Stable integration to cell genome (19)	Low efficacy (19)

DNA plasmid	Lower cost (20)	Reduced efficacy (22)
	Low immunogenicity (21)	Decreased genome integration (22)
	Decreased risk of insertional oncogenesis (21)	Early exhaustion of T cells (21)
		Limited persistence and expansion of engineered cells (20)

Messenger RNA	Transient expression of the transgene (1 week) (23)	No integration of the transgene into the cell genome (23)

**Figure 2:**
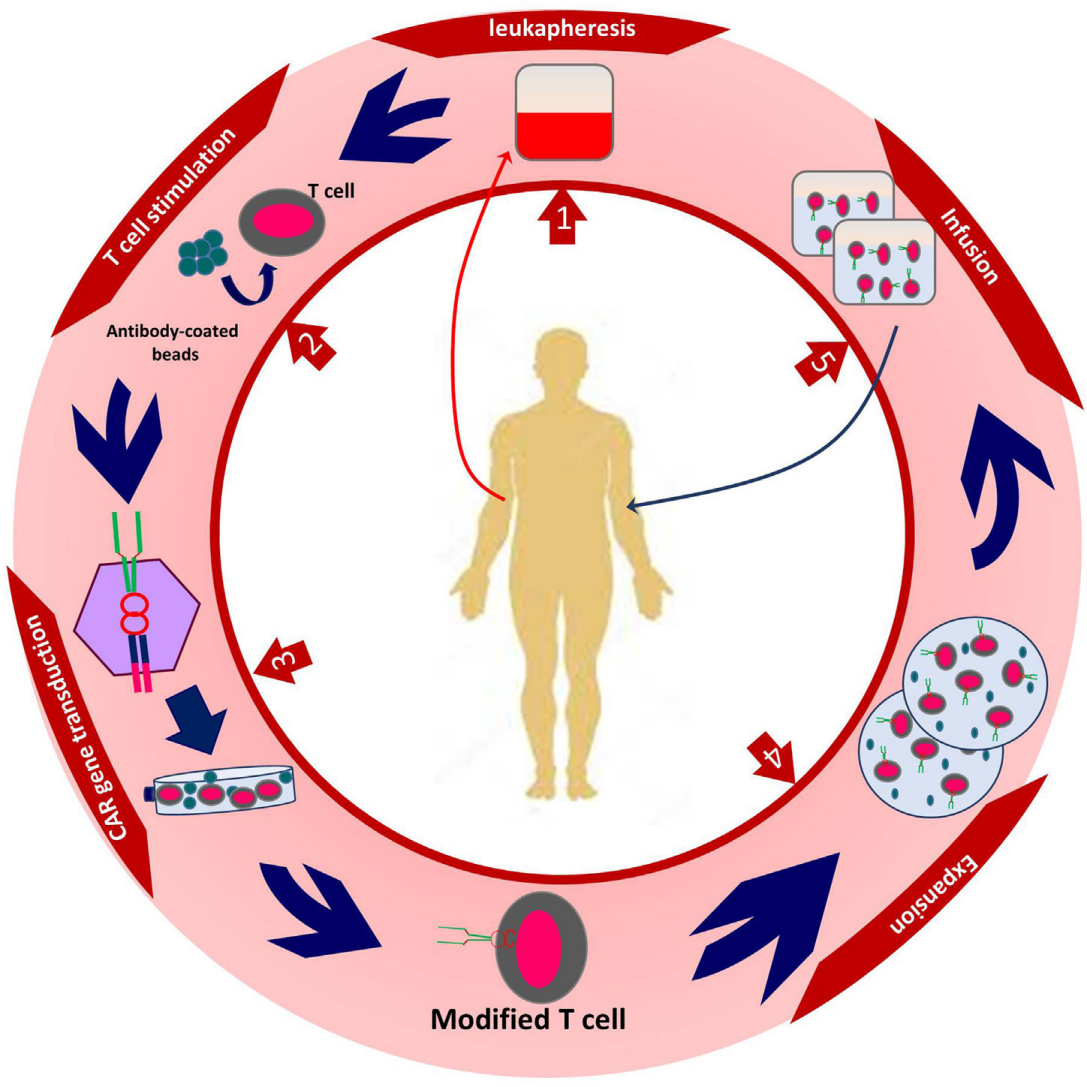
The procedure of autologous chimeric antigen receptor (CAR) T cell production. Created by Esmaeilzadeh et al.

After transduction, genetically modified T cells are cultured to reach the appropriate number in order to have expected efficacy. Different steps of CAR T cell production take about 2 weeks. The final step is to infuse genetically modified T cells to the patients (13, 24, 25) (Figure 2).

Administrating significant clinical guidelines and notifying CAR T cell therapy considerations guarantee the quality and safety of this treatment method. It is necessary to set specific and standard guidelines for the application of CAR T cells. The process of CAR T cell production can be applied both by Good Manufacturing Practices or automated manufacturing (26, 27).

In conclusion, CAR T cells are living drugs with special ability to persist and proliferate in patient’s body. The process of CAR T cell production from preclinical steps to clinical administration raises some challenges and controversies which slow down the rate of its development. The aim of this article is to determine the obstacles and forecast the upcoming novel generations of CAR T cell engineering by investigating present data from recruiting and completed clinical trials. This may provide a new insight of CAR T cell engineering and a promising window to create smarter next generation of CAR T cells.

## An Overview of Ongoing Trials and Antigen Targets

**Figure 3:**
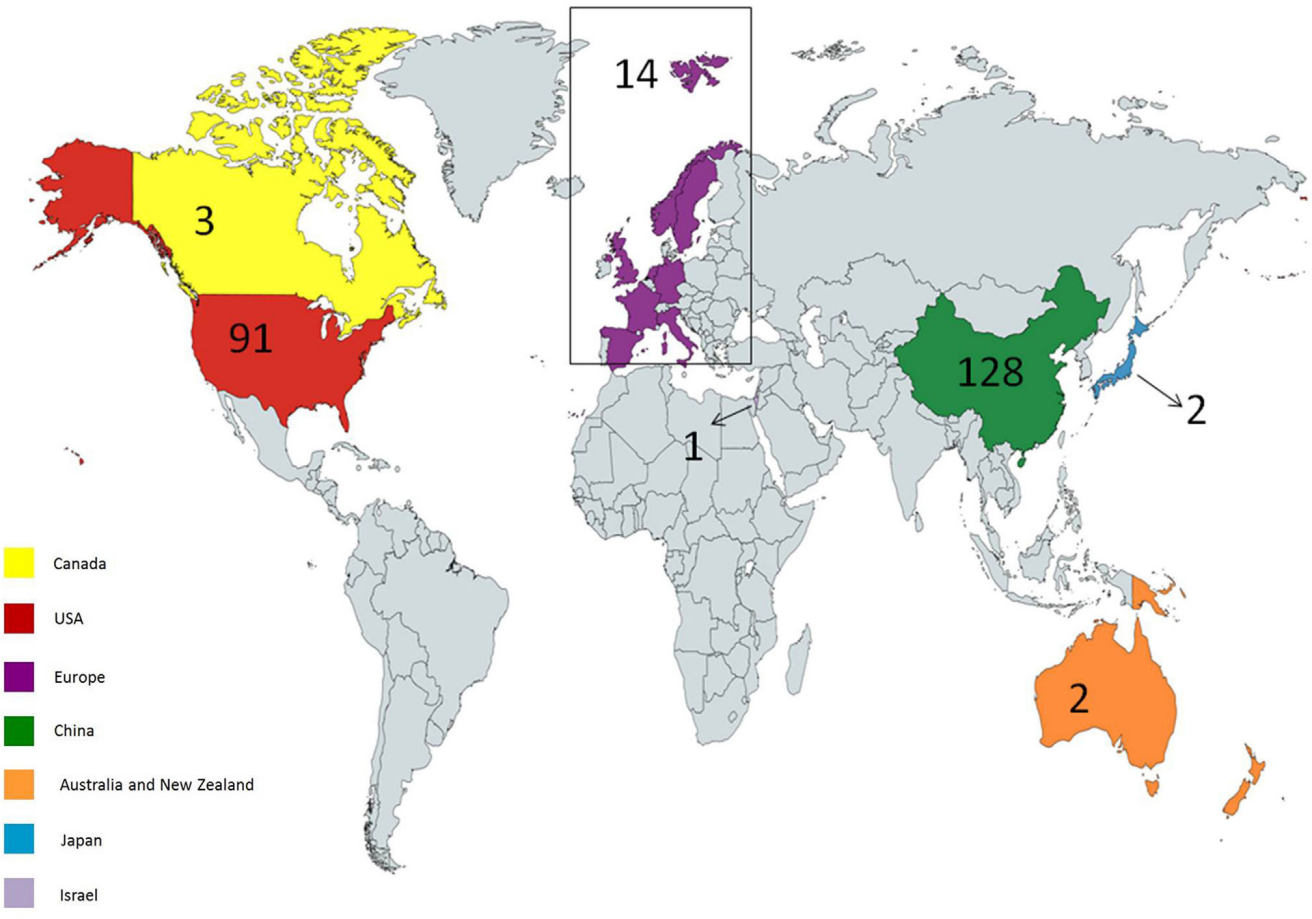
Geographical distribution of ongoing chimeric antigen receptor (CAR) T cell therapy clinical trials for cancer. USA and China hold most of the trials; however, other countries have also considered holding trials. Created by Esmaeilzadeh et al.

Up to January 2018, 241 clinical trials have been registered at clinicaltrials.gov. 171 clinical trials are ongoing, most of which focus on hematological malignancies. There are many reasons for this, such as high incidence and accessible cell surface markers of hematological malignancies. Seven clinical trials are of unknown status. Most of the studies are in phase 1 or 2, although there is one study in phase 3 (NCT03027739) and one study in phase 4 (NCT02992834). The first country to start CAR T cell trials was the USA. Other countries also commenced multiple trials thereafter. 56 trials are ongoing in the USA, 104 in East Asia, 3 in Canada, 2 in Pacifica, 1 in Japan, 13 in Europe, and 1 in the Middle East (Figure 3).

Most trials have focused on CD19^+^ B-cell hematologic malignancies such as acute lymphoblastic leukemia (ALL), lymphoma, and chronic lymphoblastic leukemia (CLL). The clinical outcome of patients treated with CD19 CAR T cells is promising, leading to complete or partial remission (PR) after treatment in many cases (28). Currently, 116 out of 238 trials target CD19. Following the success gained in CAR T cell therapy of hematological malignancies, many other clinical trials focus on both hematologic and solid tumors. Currently, 74 studies aim at solid tumors and at 161 hematological malignancies. Also, some clinical trials targeting other diseases have been designed. Currently, there are two trials aiming for HIV/AIDS (NCT03240328, NCT02471430) and one aims at systemic lupus erythematosus (SLE) (NCT03030976).

Most studies of hematological malignancies focus on CD19. However, other targets have also been studied such as CD22, CD20, IM19, BMCA/TACI, P-BMCA-101, CD13, and CD5. In solid tumors, targets include programmed death 1 (PD-1) [for epidermal growth factor receptor (EGFR) family member positive advanced solid tumors], GD2 (neuroblastoma and sarcoma), EphA2 (in glioma), AFP (hepatocellular carcinoma), ErbB2/Her2 [in human epidermal growth factor receptor 2 (HER2) positive cancers], MUC-1 (advanced refractory solid tumors), mesothelin (29), carcinoembryonic antigen (CEA) (30), prostate-specific membrane antigen (31), glypican 3 (hepatocellular carcinoma), interleukin-13Ra2 (IL-13Ra2), EGFR, EGFR variant III [recurrent glioblastoma multiform (GBM)], and VEGFR2 (metastatic melanoma) (Table 2).

**Table 2:** Information of ongoing clinical trials registered in clinicaltrials.gov.

Target antigen	Disease	Phase	Clinical trial identifier code*
**Clinical trials of hematological malignancies**			
CD19	Leukemia	1 or 2	NCT02975687, NCT03097770, NCT03208556, NCT03016377, NCT03263208, NCT03064269, NCT02924753, NCT03142646, NCT03391739
	Lymphoma	1 or 2	NCT03029338, NCT02081937, NCT03146533, NCT02842138, NCT03208556, NCT02652910
	Lymphoma and leukemia	1 or 2	NCT02819583, NCT03383952, NCT03271515, NCT03110640

CD20	Leukemia and lymphoma	1 or 2	NCT02710149

	Lymphoma	2	NCT03277729, NCT02965157

CD19 and CD20	Leukemia and lymphoma	1	NCT03097770, NCT03019055
	Lymphoma	1 or 2	NCT03207178

CD22	Leukemia and lymphoma	2	NCT02935153
	Lymphoma	1	NCT03244306

CD19 and CD22	Leukemia and lymphoma	1 or 2	NCT03233854, NCT03185494, NCT03098355

CD30	Lymphoma	1	NCT03383965, NCT03049449

BCMA	Multiple myeloma	1 or 2	NCT03287804, NCT03288493, NCT03070327, NCT03338972, NCT03322735, NCT03380039

CD123	AML	1	NCT03114670
	BPDCN	1	NCT03203369

CD33	Leukemia and lymphoma	1	NCT03126864

Ig k	Lymphoma	1	NCT00881920
	Myeloma		
	Leukemia		

ROR1	Breast	1	NCT02706392
	Lung		
	Acute lymphoblastic leukemia		
	Chronic lymphoblastic leukemia		
	Lymphoma		

**Clinical trial of solid tumors**			

EGFR	Sarcoma	1	NCT00902044
	Glioblastoma	1	NCT02442297
	Glioblastoma multiform (GBM)	1	NCT02844062
	Recurrent GBM	1	NCT03283631
	Recurrent brain tumors		
	EGFR-positive colorectal cancer	1 or 2	NCT03152435
	Advanced solid tumor	1 or 2	NCT03182816
	Advanced solid tumor	1 or 2	NCT02873390

EGFRvIII	Glioblastoma	1	NCT02664363
	GBM	1	NCT02844062
	Malignant glioma	1 or 2	NCT01454596
	Brain cancer		
	Pancreatic cancer	1	NCT03267173

HER2	HER2 positive cancers	1 or 2	NCT02713984
	Glioblastoma	1	NCT02442297
	Sarcoma	1	NCT00902044

Mesothelin	Advanced solid tumor	1 or 2	NCT03182803
	Mesothelin positive tumors	1	NCT02930993
	Advanced solid tumor	1 or 2	NCT03030001
	Pancreatic cancer	1 or 2	NCT01583686
	Cervical cancer		
	Ovarian cancer		
	Mesothelioma		
	Lung cancer		
	Cervical cancer	1 or 2	NCT03356795
	Pancreatic cancer	1	NCT03323944
	Malignant pleural disease	1	NCT02414269
	Breast cancer		
	Lung cancer		
	Mesothelioma		
	Pancreatic cancer	1	NCT02706782
	Breast cancer	1	NCT02792114
	Hepatocellular	1 or 2	NCT02959151
	Pancreatic cancer metastatic		
	Colorectal cancer metastatic		

PSMA	Cervical cancer	1 or 2	NCT03356795
	Urothelial bladder carcinoma	1 or 2	NCT03185468
	Bladder cancer		
	Prostate cancer	1	NCT03089203

CD70	Pancreatic cancer	1 or 2	NCT02830724
	Breast cancer		
	Ovarian cancer		
	Renal cell cancer		
	Melanoma		

MUC1	Lung cancer	1	NCT03198052
	Non-small cell lung cancer	1 or 2	NCT02587689
	Triple-negative invasive breast carcinoma		
	Hepatocellular carcinoma		
	Pancreatic carcinoma		
	Advanced solid tumor	1 or 2	NCT03179007
	Gastric carcinoma	1 or 2	NCT02617134
	Colorectal carcinoma		
	Malignant glioma of brain		
	Lung cancer	1 or 2	NCT03356808
	Cervical cancer	1 or 2	NCT03356795
	Sarcoma	1 or 2	NCT03356782
	Osteoid sarcoma		
	Ewing sarcoma		
	Pancreatic cancer	1	NCT03267173

GD2	Neuroblastoma	1 or 2	NCT03373097
	Neuroblastoma	1 or 2	NCT02765243
	Cervical cancer	1 or 2	NCT03356795
	Relapsed or refractory neuroblastoma	1	NCT02761915

CEA	Colorectal cancer	1	NCT02349724
	Breast cancer		
	Lung cancer		
	Pancreatic cancer		
	Gastric cancer		

GPC3	Lung squamous cell carcinoma	1	NCT02876978
	Hepatocellular carcinoma	1	NCT03198546
	Squamous cell lung cancer		
	Hepatocellular carcinoma	–	NCT03146234
	Hepatocellular carcinoma	1 or 2	NCT03130712
	Hepatocellular carcinoma	1 or 2	NCT02715362

MET	Malignant melanoma	1	NCT03060356
	Breast cancer		

PD-L1	Non-small cell lung cancer	1	NCT03060343
	Advanced lung cancer	1	NCT03330834
	Lung cancer	1	NCT03198052

*BMCA, B-cell maturation antigen; Ig, immunoglobin; ROR1, receptor tyrosine kinase-like orphan receptor; MUC, mucin; EGFRvIII, EGFR variant III; EGFR, epidermal growth factor receptor; HER2, human epidermal growth factor receptor 2; GPC3, glypican 3; CEA, carcinoembryonic antigen; PSMA, prostate-specific membrane antigen; PD-L1, programmed death-ligand 1; PD-1, programmed death-1*.

*Data of ongoing clinical trials are confirmed by clinicaltrials.gov.

## Clinical Advantages of CAR T Cell Therapy in Malignancies

### Advantages of CAR T Cell Therapy in Hematological Malignancies

CD19 is a specific marker of B-cell lineage, not being expressed on other cell lines, and thus could be an attractive target for engineering T cells against several B-cell hematological malignancies (32). Considerable clinical responses of relapsed/refractory (R/R) B-ALL treatment with CD19 CAR T cells have been reported. There are several groups informing complete remission (CR) rate up to 90% in CD19 CAR T cell therapy of R/R B-ALL patients (33–35). Also, some studies have reported the efficacy of CD19 CAR T cells in lymphoma patients, which led to more than 40% response in diffuse large B cell lymphoma and more than 70% in indolent lymphoma (36). In a survey documented in 2015, CD19 CAR T cell therapy in multiple myeloma led to the eradication of the disease after 12 months (37, 38). Also, targeting five patients of CD138^+^ multiple myeloma led to stable disease state in four of them (39). In CLL, around 50% of patients treated with CAR T cells experienced remission for more than 5 years of infusion (40). Another clinical trial enrolled four CLL patients in which three of them exhibited CR and one resulted in PR (6).

CD33 is a surface antigen presented by more than 80% of the AML malignant cells, but also is expressed on normal myeloid progenitor cell lines. Preclinical promising data proposed CD33 CARs to be employed for clinical use. In a relapsed and refractory acute myeloid leukemia patient, CAR T cells reported to induce a short time benefit related to the high levels of cytokines in the patient’s blood; however, substantial adverse effects including fever and fluctuations in the pancytopenia were later reported (41).

As mentioned above, CD19 is the most targeted antigen in hematological malignancies; however, sometimes B cells lose the expression of CD19 on their surface. This would lead to the resistance to CD19 CAR T therapy. In a phase 1 study in 2018, B-ALL patients who were resistant to CD19 CAR T cell therapy were treated with CD22 CAR T cells. This study demonstrated a biologic dose-dependent anti-tumor activity of CARs in an antigen density-dependent manner (42). CD20 can be targeted in different diseases especially lymphoma. In 2016, a phase 2 trial administrated anti-CD20 CAR T cells for refractory/relapsed CD20^+^ B-cell non-Hodgkin’s lymphoma patients. From 11 patients, 3 patients exhibited partial responses and 6 experienced CRs. Also, no substantial toxicities were reported. This study also revealed that the underlying reason for the differences between disease progression and the patient may be related to the levels of CAR gene (43). Clinical advantage of engineering CAR T cells against other antigens of hematological malignancies is still being investigated.

### Exploitation of CAR T Cells Against Solid Tumors

In contrast with impressive outcomes achieved using CAR T cells against hematological malignancies, results in solid tumors seem to be less favorable. This which may be related to the immunosuppressive microenvironment of solid tumors, reduced potency of CAR T cells in order for trafficking to tumor environment, the absence of appropriate tumor-associated antigens (TAAs) and the risk of side effects especially on-target off-tumor (44). Hostile tumor microenvironment contributes to decrease the optimum efficacy of CAR T cells *via* multiple mechanisms such as the activity of fibroblasts and extracellular matrix, soluble factors/cytokines (such as TGFβ), and immunosuppressive immune cells including T-regs and myeloid-derived suppressor cells (MDSCs) (45). Thus, multiple novel approaches need to be designed to improve the efficacy of these cells.

In order to bring the benefit of CAR T cells to the clinic, some *in vitro* studies were performed which demonstrated their efficacy on multiple solid cancer cell lines. In this article, we focus on the clinical administration of CARs, especially on patients. Multiple solid malignancies have been targeted by CAR T cells. One important step is the recognition of appropriate tumor antigen that is highly and specifically expressed on tumor cells. Epidermal growth factor receptor (EGFR) is expressed by more than 50% of non-small cell lung carcinoma cells and thus may a good candidate. In 2016, Feng et al. (46) evaluated the efficacy and safety of EGFR-CAR T cells in 11 patients. The CAR T cells were infused in multiple doses. This study reported two patients to experience partial response and five patients experienced stable disease.

Human epidermal growth factor receptor 2 is a cell surface antigen presented on several cancers including breast, ovarian, GBM, and medulloblastoma. There are some studies reporting the preclinical efficacy of CAR T cells in HER2^+^ GBM, ovarian breast, osteosarcoma, and medulloblastoma of orthotopic xenogeneic models (47–51). A phase 1 clinical trial assessed the benefit of HER2-specific CAR T cells for HER2^+^ sarcoma. The infused T cells reported persisting at least 6 weeks in seven patients of nine who were evaluable. Also, in three patients, the tumor was reported to remove with more than 9% necrosis. This study exhibited considerable tumor eradication and anti-tumor activity with no evident toxicities in patients (52). There are several other ongoing trials targeting multiple TAAs in different solid tumors such as mesothelin, IL-13Rα2, and CEA.

An important part of the limited efficacy of CAR T cells against solid tumors is related to the immunosuppressive tumor microenvironment. This hurdle can be overcome by administration of the transgene encoding IL-12 by the T cells. In 2015, a phase 1 study targeted six recurrent MUC16^ecto+^ ovarian carcinoma patients with armored IL-12 secreting CAR T cells. The selection of an appropriate TAA along with the secretion of IL-12 by T cells led to the enhanced persistence of the CAR T cells. Also, the expression of the IL-12 appropriately modulated the tumor microenvironment and increased the cytotoxicity of the cells (53, 54).

Several trials have targeted different solid cancers and variable results have been achieved; however, more modifications and engineering approaches are required to improve the advantage of CAR T cell therapy in solid tumors.

## Side Effect and Toxicity

Although excellent results have been achieved in CAR T cell therapy trials, they can also be accompanied by some adverse effects. CAR T cell infusion may even cause some life-threatening toxicities (44). Some of these side effects are discussed here.

### Cytokine Release Syndrome (CRS)

Cytokine release syndrome is the most prevalent toxicity observed after infusion of engineered T cells. Its occurrence is related to the intense activation of the infused T cells which activate other immune cells; altogether, producing the extended amount of cytokines resulting in a cytokine storm (55). CRS is so dangerous since it can cause fever, nausea, fatigue, myalgia, vascular leakage, hypotension, and multiple organ failures. Even death can be accompanied by CRS following infusion of CAR T cells (56). Multiple organs and systems including cardiovascular, pulmonary, renal, hepatic, musculoskeletal, and hematologic systems may also be involved in CRS. The predisposition factors for CRS are high tumor burden as well as the high dosage of infused CAR T cells. Also, stronger propagation and activation of T cells increases the risk of CRS (57). Recently, some serum biomarkers have been introduced as predictive biomarkers for CRS such as high levels of CRP (over 20), IL-6, and IFNγ (58).

### On-Target Off-Tumor Toxicity

On-target off-tumor toxicity occurs when T cells lose the ability to distinguish normal cells from tumor cells. TAAs are the most antigens employed for the production of CAR T cells and are expressed on both tumor and normal cells. Attacking normal cells expressing TAA leads to the destruction of them named as on-target off-tumor toxicity (59).

The most common form of on-target off-tumor toxicity is the destruction of B-cells which leads to B-cell aplasia. This is commonly observed in CD19 CAR T cell therapies (60). Since HER antigen is expressed by cardiac and pulmonary epithelial cells, HER2 CAR T cells applied for breast cancer can exhibit cardiopulmonary toxicity (48). Based on a case report in 2010, ERBB2 CAR T cell therapy for colorectal cancer led to the death of the patient because of pulmonary toxicity (56).

In order to reduce the range of this toxicity, more specific antigens such as tumor-specific antigens (TSAs), only expressed on tumor cells are preferred for CAR T cell designing.

### Neurotoxicity

Neurotoxicity has been observed in some trials, which can be a result of T-mediated inflammation, elevated cytokine levels in CNS and cerebral edema; however, the exact reason has not been known yet. Symptoms of neurotoxicity of CAR T cells include aphasia, confusion, delirium, word finding difficulty, myoclonus, and seizure (35, 59). Hu et al. reported an R/R ALL female who experienced neurological symptoms because of cerebral CRS, 6 h and 3 days after transfusion of the autologous CAR T cells. The patient was then treated with methylprednisone until day 14 which diminished patient’s symptoms completely (61). Although most centers have reported neurotoxicity to be self-limited with no long-term neurologic deficits, some death cases have been reported related to the neurotoxicity caused by cerebral edema (43). At present, there are no standard clinical interventions for the management of the neurotoxicity, but systemic corticosteroids may be employed in case of severe side effects. Also, dexamethasone may be chosen due to its heavy penetration into the CNS (62).

### Anaphylaxis

Anaphylaxis is a kind of immediate toxicity related to the immunogenicity caused by murine antibody derived ScFV. Humanizing components of the CAR protein may reduce the chance of anaphylaxis, which has been discussed in further sections of this article (51). Since anaphylaxis is lethal, instant treatment of this life-threatening toxicity is necessary for the patients (59, 63).

### Tumor Lysis Syndrome (TLS)

Tumor lysis syndrome is also one of the toxicities correlated with sudden tumor cell death and is defined by increased lactate dehydrogenase, uric acid, and potassium levels. TLS may also result in acute kidney injury. Decreasing the size of the tumor before infusion, patient intravenous hydration, and Rasburicase treatment may be helpful to reduce the severity of TLS (59).

### Insertional Oncogenesis

Insertional oncogenesis is related to the transfer of a retroviral or lentiviral transgene to the T cells. This may be accompanied by a higher risk of malignancy induction in the target cell. The probability of oncogenesis followed by transduction seems to be low; however, more precautions and monitoring strategies will be required in future clinical trials (59, 64–66).

## New Generations of CAR T Cells

Four generations of CAR T cells have been produced yet; however, more modifications and developments are still in process to enhance their clinical advantage and efficacy.

### Developing CAR T Cells With Clustered Regularly Interspaced Short Palindromic Repeats (CRISPR)/CRISPR-Associated 9 (Cas9)

Clustered regularly interspaced short palindromic repeats/Cas9 is a novel gene manipulation technology system with a great potential for biologic genome editing processes. Administration of CRISPR/Cas9 as a novel technology for genome editing could help to design more effective therapeutic agents (67). Recently, application of CRISPR/Cas9 in combination with cancer immunotherapy has been introduced to construct the next generation of CAR T cells. Rupp et al. employed Cas9 ribonucleoprotein (Cas9 RNP) as the gene manipulation method *via* lentiviral transfer in order to produce CD19 CAR T cells insufficient of PD-1. PD-1 is a member of immune checkpoint inhibitor family. The expression of the ligand for PD-1, named PD-L1, on tumor cells can reduce the anti-tumor function of T cells and decrease their optimal activation. Disrupting the gene responsible for the expression of the PD-1 in T cells, *Pdcd1*, by CRISPR/Cas9, leads to the absence of PD-1 cell surface. This modification inhibits the anti-immune checkpoint inhibitory function of the tumor cells and has shown to increase the demolition of the PD-L1^+^ xenograft *in vivo* tumor model (68).

These data demonstrate that genome editing using CRISPR/Cas9 system may be employed to produce next generation of modified CAR T cells by genome editing of more immune checkpoints, surface antigens, and secretory enzymes, and cytokines to enhance the clinical therapeutic effects.

### Designing New Targets

Although multiple approaches have been utilized to improve the anti-tumor potency of CAR T cells, selection of an optimal target antigen for the production of CAR would pave the road to produce a new generation of CAR T cells. ScFV CARs have illustrated impressive results in CAR T cell therapy for both hematological and solid malignancies; however, off-target toxicity, low specificity, and immunogenicity still remain as challenges (69, 70). Designing human origin ScFV-based CARs such as M28z CAR, a new CAR containing m912, led to the initiation of a clinical trial targeting mesothelioma, lung, and breast cancer (NCT02414269). Thus, humanizing the origin of ScFV can reduce the immunogenicity of CAR design.

Targeting intracellular antigens such as Wilms Tumor 1 (WT1) in cancer could provide a new road to target antigen selection (71). Rafiq et al. engineered a T-cell receptor-mimic CAR to target the intracellular oncoprotein named as WT1, which is also expressed on the cell surface through HLA-A*02:01. These engineered WT1-28z specific CAR T cells destroyed and lysed HLA-A*02:01, WT1^+^ tumor cells in an *in vivo* mice model (71).

Single chain Variable Fragment antibody formats with lower side effects, improved affinity, and specificity will make utilization of CARs more favorable for CAR T cell therapy. Novel antibody pieces such as single domain VH, VHH, mini, dia, and triabody have recently been introduced (72, 73). An example is the GPA7-28z redirected TCR-like CAR-engineered against melanoma. The ScFV domain was obtained from Lamma-derived VHH part of the antibody against melanoma cells. GPA7-28z engineered T cells possessed enhanced cytotoxic characteristics when administrated both *in vitro*, against human melanoma cells, and *in vivo*, against xenograft model (74).

Cancer stem cells (CSCs) are present in most cancers and are responsible for relapse and resistance to therapy. These cells can be distinguished from other cancer cells by specific surface antigens such as CD34, 44, 90, 133, and EpCAM (75). Targeting CSC target antigens as CAR T cell targets may provide a potential opportunity to enhance the clinical response by eliminating them. EpCAM^+^ prostate CSCs can be an appropriate target for EpCAM-specific CAR T cells since they have an important role in tumor proliferation and progression. In a study in 2015, EpCAM-redirected CAR T cells were employed against EpCAM^+^ prostate cancer in both *in vitro* and *in vivo* models. EpCAM-specific CAR T cells exhibited substantial anti-tumor toxicity against prostate metastatic tumor cells (76, 77).

Also, one of the major challenges that limit the function of CARs against solid tumors is the heterogeneity of tumor antigens. It is recently demonstrated that tumor cells express cancer-specific cell surface antigens that are caused by post-translational alterations of the antigens. Mostly, O-glycosylation of the antigens leads to the expression of these cancer-specific targets named as glycan-antigens. Anti-Glycan CAR T cells are the novel approach employed for the construction of the CARs that are highly specifically redirected against special tumor surface antigens. Different antibody classes, such as high-affinity O-glycopeptide antibodies, may be employed for the construction of glycan-CARs. Using glycan targets for designing CARs can emerge as a powerful tool for precisely targeting solid tumor cells (78).

The fast-growing wave of CAR T cell therapy needs the application of new unique target antigen strategies to engineering the next generations, which would lead to better clinical outcomes and fewer side effects.

### Modification of TRUCK T Cells

TRUCK T cells or fourth generation of CAR T cells encompass the particular capability of delivering a transgenic material (payload) to the tumor site. To achieve this, T cells are engineered with a nuclear factor of activated T cell (NFAT), which is responsible for the expression of the transgenic product (such as cytokines). IL-12 is the most studied cytokine secreted by the transgene part of the CAR T cells. Production of pro-inflammatory cytokine (such as IL-12) by CAR T cells can activate both innate and adaptive immune system which leads to a robust anti-tumor immune activity. Also, IL-12 can inhibit the immune suppressor activity of myeloid-derived suppressor cells as well as regulatory T cells. CAR T cells secreting IL-12 possess enhanced anti-tumor function and improved therapeutic effect, especially when administered against solid tumors (79).

Although TRUCK T cells have shown impressive results in clinical trials, controlling the titer of the cytokine secretion must be considered. This is due to the possible toxicities in case of severe cytokine production related to the elevated amount of IFNγ. Uncontrolled secretion of IL-12 would lead to severe side effects damaging lung, liver, and bone marrow. The solution to this problem is to adjust the strength of the promoter or to administrate the NFAT to engineer promoters (80). In order to benefit this technique, two ongoing phase 1/2 clinical trials by National Institutes of Health Clinical Center have engineered TCR T cells or TILs, with the ability of cytokine secretion (IL-12), to target NY-ESO-1^+^ solid tumors (NCT01457131, NCT01236573). However, new methods that help to regulate the secretion of cytokines by TRUCK T cells, such as regulating the gene responsible for expression of the cytokine using 1miRNAs or siRNAs, are required. Also, equipping TRUCK T cells with the ability to secrete enzymes (such as heparanase) that ameliorate their infiltration to the tumor stroma can increase the number of infiltrated CAR T cells to the solid tumor site and thus can increase the anti-tumor efficacy of these cells.

In a study in 2016, the genome of the allogeneic universal CAR T cells was edited with electroporated CRISPR/Cas9 to render them resistant to PD-1 inhibition *via* editing its gene on T cells. These manipulated universal T cells decreased alloreactivity and increased the anti-tumor potential of CAR T cells (81).

Altogether, application of novel genome editing methods and strategies along with improved safety approaches may help to modify TRUCKs in through more effective outcome.

## Building Smarter CAR T Cells to Increase Therapeutic Efficacy and Reduce Adverse Effects Especially in Solid Tumors

**Figure 4:**
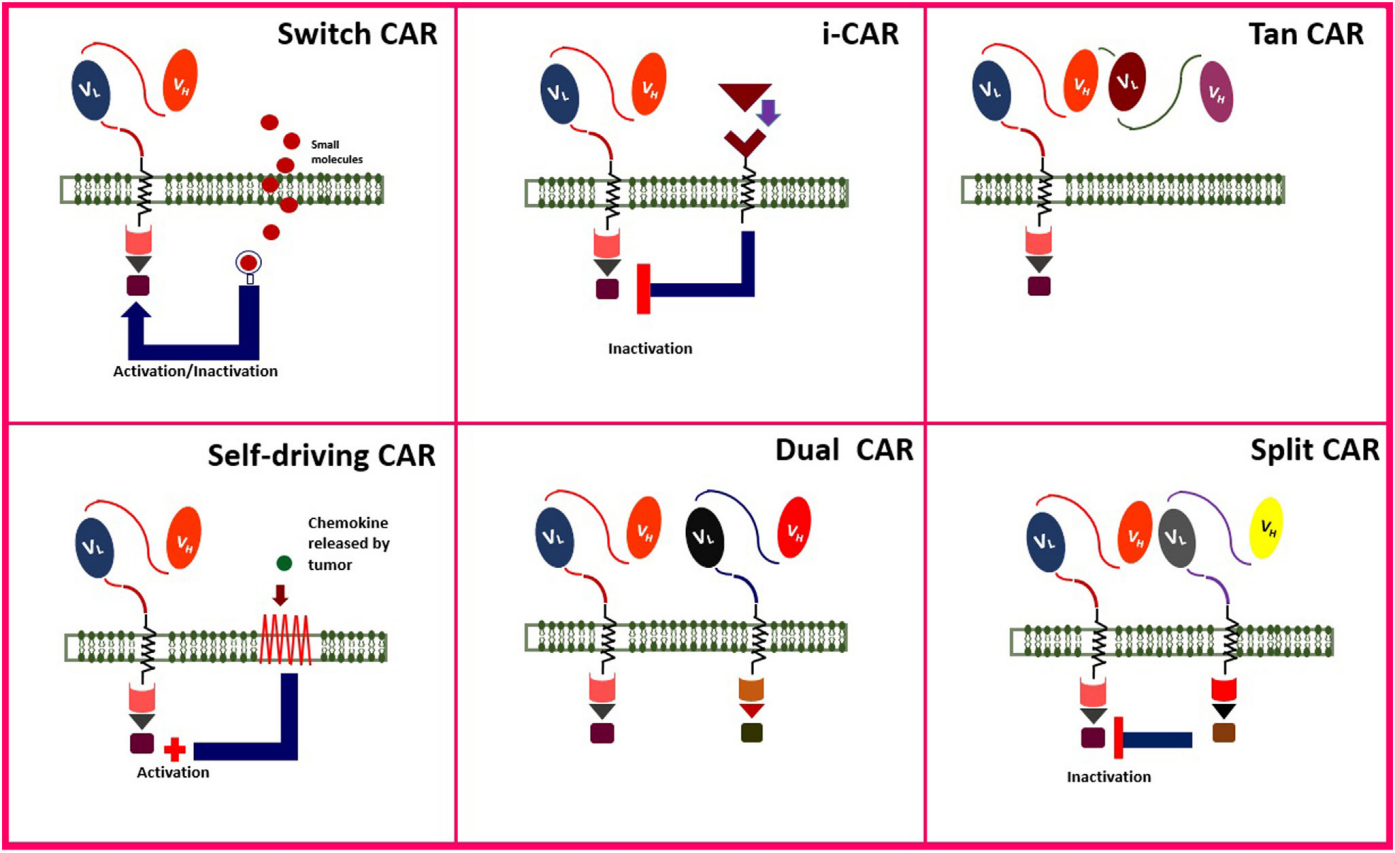
Novel modified chimeric antigen receptor (CAR) designs. Created by Esmaeilzadeh et al.

Adoptive cell therapy, especially CAR T cell therapy, is a combination of immune, gene, and cell therapy (82, 83). Engineering smarter CAR T cells improve the strength, quality, safety, efficacy, and anti-tumor function of the genetically engineered cells. Administration of CAR T cells for their anti-tumor benefit is accompanied by some adverse effects, as well. Some of the novel generations of CAR T cells are addressed in **Figure 4**.

One method to improve the safety and manipulate the function of genetically engineered T cells is to control them by adding “switches.” This strategy makes it possible to induce death or inactivate the T cells by adding an exogenous component (off-switch). The example of off-switch is the administration of Herpes Simplex Virus thymidine kinase (HSV-TK) or inducible caspase-9 (iCasp-9) as self-destruction genes, which could be activated by injection of ganciclovir and FK506 binding protein, respectively. This approach makes it possible to control the activity of CAR T cells and even to finish their activity in case of substantial toxicity. Activation of HSV-TK (84) and iCasp-9 (85) would induce the apoptosis of the T cell and cessation of its activity in order to decrease its side effects. As an example, Gargett and Brown engineered the third generation of GD-2-redirected CAR T cells with iCasp-9 against melanoma tumor cells (85).

“On-switch” CAR T cell is another approach in which an exogenous molecule can induce the activation of engineered T cells (86). On-switch has some advantages than the off-switch method; e.g., on-switch T cells are not necessarily destroyed in the absence of the exogenous component, as well as being safer than off-switch method. However, frequent administration of the activating molecule may lead to resistance of the patient to therapy (87).

Lim et al. described another targeting system based on synthetic NOTCH receptors, responsible for sensing/response behaviors, which are expressed only when it is linked to a tissue-specific ligand (88).

Inhibitory CAR (iCAR) is a new method which can be administrated to inhibit CAR T cells in case of severe toxicities. The T cell surface domain includes a receptor for PD-1 and cytotoxic T lymphocyte-associated antigen 4 (CTLA-4), as normal immune checkpoint inhibitor molecules. Receptors of these immune checkpoints are expressed on T cells. Blocking these checkpoints would lead to the inactivation of the immune cell. Thus, it was proposed to inactivate CAR T cells by infusion of PD-1 or CTLA-4 in case of severe toxicities (89, 90).

Also, T cells can be engineered to express the CAR transiently (self-limiting CARs). Self-limiting CARs are automatically inactivated and destructed after the transient expression of CAR is over. When the functional period of CAR is over, T cells will return to their normal function in the tissue. In order for transient expression of CAR, transposon vectors are employed as gene transduction strategies (91).

Engineering T cells marked with monoclonal antibody (mAb) as cell surface antigen could launch the anti-tumor function of the cell, just in case, it is linked to the tumor cells expressing the antigen. Two examples of this strategy include the application of CD34/CD20 (92) and EGFR (93) on CAR T cells which can be eliminated by rituximab (anti-CD20 mAb) and cetuximab (anti-EGFR mAb) in case of severe toxicities. Philip et al. (92) engineered highly sensitive CD37 and CD20 antigens and administrated rituximab for selective elimination of the genetically transformed cells.

Tandem CAR (TanCAR) is another method of genetically engineering T cells in order to reduce the off-tumor toxicity. TanCAR contains two different kinds of ScFVs and is activated only if both antigens are introduced to the T cell. HER2^+^/CD19 and HER2^+/^IL-13Rα2 are two examples of TanCARs which have been studied on glioblastoma tumor expressing both antigens (94, 95). TanCARs can high specifically exhibit anti-tumor toxicity against tumor cells since both antigens must be present on the tumor cell surface. This specificity would increase the safety of TanCARs than conventional CARs that were only specific against one cell surface antigen.

In order to increase the precision of CAR T cells to target only cancer cells in the patient, it was proposed to concurrently engineer CAR specific against two tumor antigen named as dual CARs. Dual CARs are composed of two CARs, containing both primary signaling (CD3ζ) and secondary signaling (co-stimulatory) domains. Accumulating data suggest that simultaneous targeting of two tumor antigens can enhance the accuracy of tumor cell distinguish and empower the anti-tumor activity of CAR T cells. Also, since two tumor antigens are targeted by dual CARs, the risk of on-target, off-tumor toxicity is also reduced. In 2013, a dual mesothelin-FRaCAR coexpressing both signal 1 (anti-Meso ScFV-CD3z) and signal 2 (anti-FRa ScFV-CD28) was engineered. In this study, CAR T cells exhibited enhanced cytokine secretion only against tumor cells that expressed both antigens simultaneously, but not against cells that contained one of the antigens. These CAR T cells also involved increased *in vivo* persistence and anti-tumor activity (96). In another study, breast cancer cells were treated with HER2-mucin 1 (MUC1) CAR T cells which exhibited enhanced complementary signaling along with increased precise cytotoxicity against breast cancer cells expressing both antigens (97).

Tandem CARs and DualCARs are similar in terms of their mechanism since they both target two antigens on tumor cells; however, their mechanism of target recognition is distinct. Also, both of these strategies have reported reducing antigen escape, which is a major cause of resistance to therapy in cancer. This leads to the enhanced anti-tumor activity of CAR T cells against cancer (98).

Immunosuppressor agents infused before CD19 CAR T therapy along with B cell aplasia followed by injection of CD19 CAR T cells can lead to the invasive fungal infection (IFI), especially by *Aspergillus* and *Candida*, in patients. To solve this problem, it was proposed to engineer dual CD19-Dectin-1 CAR targeting both malignant B cells and fungal hyphae. This approach has reported to successfully inhibit the IFI after CAR-19 therapy for leukemia and lymphoma patients (99).

Decision-making CAR T cells, smart T cells activated only in a special condition, have recently been designed. These CAR T cells are engineered in a way that they are activated in the patient’s body only if special environment such as hypoxia is prepared. Since hypoxia is a special characteristic of the tumor microenvironment, oxygen-sensitized CAR T cells were designed. These CAR T cells initiate their anti-tumor function only in hypoxic condition. As normal cells do not typically experience hypoxia, this prevents CAR T cells to be activated in the microenvironment of the normal cells and can thus decrease the officious side effects. Hypoxia-inducible factor (HIF) is a molecule produced in hypoxic tissues and is overexpressed by cancer cells. To engineer self-decision-making CARs, the ScFV domain was derived from an anti-HIF antibody. This enabled CAR T cells to traffic to the tumor site with a high specificity and not haring other normal tissues at the same time (100). This study paved the way to engineering CAR T cells with the potential for designing novel tumor-specific CAR T cells.

One important obstacle that inhibits the appropriate function of the CAR T cells is the limited homing of the CAR T cells to the tumor microenvironment. One solution is to modify CAR T cells in a way that they target tumor vessels. VEGF is overexpressed by tumor cells and increases the angiogenesis of the tumor leading to the production of vessels. In order to improve the homing of the CAR T cells to the tumor site, VEGFR targeted TRUCK T cells with the capability of IL-12 secretion have been introduced to aim the blood vessels of the tumor and have shown increased immigration of the cells to the tumor site (101, 102). Also, another approach to enhance CAR T cell delivery to the solid tumor site is to modify CAR T cells using echistatin which can strongly bind to αvβ3, a marker that is expressed by tumor vessel endothelial cells. This approach has been studied previously and promising results have been achieved (103), but more considerations need to be applied in further clinical trials.

One strategy to reduce the side effects caused by CAR T cells is to convert the progression and activation of CAR T cells dependent on specific adaptor molecules. It is recently proposed to modify antibodies (e.g., CD19 and CD22) produced for CAR production with fluorescein isothiocyanate (FITC). This strategy provides the feasibility of controlling the precise function and geometry of FITC-CAR T cells. Introducing the receptors of FITC to T cells would increase the potential anti-tumor activity and improve the trafficking of the CAR T cells to the tumor site and (104). Such targeting strategies may develop the anti-tumor potency, trafficking, and specificity of the CAR T cells.

Combinatorial immunotherapeutic approaches incorporate an immune checkpoint blockade with engineering T cells. one of the most important and effective immune checkpoint inhibitor molecules that hinders the function of the CAR T cells in tumor microenvironment is the program death that PD-1 negative CAR is designed to remain in the PD-L1 positive solid tumor microenvironment without limitation, which ameliorates the anti-tumor efficacy of CAR T cells (105).

Understanding the specific properties of the solid tumor microenvironment have provided new opportunities for modifying CAR T cells. One of the important characteristics of the solid tumor is the specific metabolism of the solid tumor which is distinct from the metabolism of the normal cells. Solid tumor metabolism leads to the production of molecules and proteins that promote its progression and angiogenesis. One important factor is the extracellular adenosine which is produced from ATP by CD73 and CD39. Adenosine promotes the angiogenesis, metastasis, and progression of the tumor cells and thus has been introduced as a promising target. Some studies have attempted to inhibit the function of adenosine either by inhibiting its receptor named as adenosine 2A receptor or its producer, CD73. These data can be as templates for designing CAR T cells that inhibit the adenosine function in clinical trials (106).

Indolamine-2,3 dioxygenase (IDO) is another tumor-associated molecule which is the catalyst for the degradation of the tryptophan, an amino acid that is necessary for the survival of T cells. High levels of IDO are produced by tumor cells and MDSCs. Inhibiting the function of IDO, *via* IDO-inhibitors, could increase the function of CAR-redirected cell therapy in clinical trials (89). Also, tumor microenvironment has known to contain high levels of anti-oxidant agents including H_2_O_2_, which can disturb the function of CAR T cells. In order to decrease the effect of H_2_O_2_ in the tumor, scientists added the ability of catalase production to the CAR T cells (CAT-CAR). Catalase producing CAR T cells were reported to possess increased resistance against the oxidative stress. Also, they were accompanied by enhanced anti-tumor activity in the tumor site (107).

Since tumor stroma is one of the major obstacles that limit the penetration of the CAR T cells to the tumor site, scientists thought to engineer CAR T cells against the fibroblast-associated protein (FAP), which is highly expressed by tumor-associated fibroblasts. These CAR T cells are named FAP-CARs. FAP-CAR T cells have been reported to increase the anti-tumor response and the survival of the xenograft models (108, 109), which has introduced it as an appropriate strategy for clinical trials (NCT01722149).

One of the limiting factors of the tumor is the immunosuppressive cytokine network including TGF-β, IL-1, and IL-4, which are present in the tumor site. Since these anti-immune soluble factors can hamper the function of T cells, inhibiting these anti-immune pathways could increase the efficacy of CAR T cells. Based on these data, Mohammed et al. engineered CAR T cells against prostate stem cell antigen (PSCA-CAR) which were modified by fusion of the IL-4 exodomain to the IL-7 endodomain (4/7 ICR CAR). This novel modification of CAR T cell was named as 4/7 ICR CAR-PSCA and was reported to increase the anti-tumor cytotoxicity of CAR T cells in the tumor site (110).

Although favorable efforts have been made to design smarter CAR T cells, more genetic and structural modifications of CAR may be helpful to increase the applicability and clinical outcomes of this adoptive immunotherapy approach. Since solid tumor microenvironment is the limiting factor for optimal function of CAR T cell therapy, it seems that further efforts must focus to overcome the microenvironment immunosuppressor effects.

## Clinical Considerations

Combining CAR T cell therapy with other immunotherapy methods such as immune checkpoint inhibitors, cytotoxic agents, and hematopoietic stem cell transplant (HSCT) may lead to better clinical outcomes. Concomitant treatment of cancer using CAR T cells and checkpoint inhibitors that block PD-1, the ligand for PD-1 (PD-L1), and CTLA-4 has exhibited great efficiency in preclinical stages. It was demonstrated that blockage of PD-1 may reinforce the CAR-T cell therapeutic effects (111, 112).

Integration of chemotherapy as lymphodepletion regimen with CART cells may improve its clinical efficacy. This may be related to multiple mechanisms such as downregulation of regulatory T cells, depletion of interfering leukocytes and decrease in tumor burden after chemotherapy. Also, diminution of CAR T cell toxicities especially CRS has been seen in the usage of this method (113). The most applied agent as lymphodepletion regimen is cyclophosphamide; although, other chemotherapy regimens such as fludarabine, doxorubicin, and platinum-based chemotherapeutics can also be administered.

Also, allogeneic HSCT can improve the therapeutic potential of CAR T cell therapy. The optimum time for the infusion of CAR T cells is 55–200 days following HSCT (114, 115).

The best outcomes may be achieved by combining cell therapy methods with routine and standard treatment strategies (115).

### CAR T Cell Dosage Modification

In order to improve the efficacy and lower the toxicities induced by CAR T cells, injection dose is divided into multiple doses. To obtain expected outcomes, the dosage of CAR T cells must reach 7.5 × 10^7^ to 3.4 × 10^8^. Of note, the number of T cells containing CAR determines the entire number of infused cells. Different centers utilize multiple infusion courses and the optimal time lapse between infusions still remains controversial (116).

### Infusion Method

After all processes of CAR T cell manufacturing, the whole volume of the product may raise to 5 l. This volume cannot be infused to the patient in one step, thus it must be divided into volumes that could be infusible (117). Different methods have been examined in order for injection of CAR T cells to the patients. Brown et al. carried out a study which exhibited 77% decrease in intracranial and spinal Glioblastoma mass lesions in intraventricular infusion method in 33 weeks. The most prevalent approach is an intravenous injection. Other infusion methods include catheter infusion, hepatic artery infusion, ultrasound-guided intratumoral injection, intraperitoneal, and intrapleural (118).

### Product Quality Control

The quality of the CAR T cell product affects its clinical efficacy which depends on the quality of donor cells, additional reagents and substances, manufacturing protocols, and CAR T cell production environment. Quality control tests are currently documented for CAR T cell clinical trials. These tests aim to focus on safety (e.g., Gram stain, mycoplasma, and endotoxin), identity (% CAR T cells), sterility, purity (e.g., %CD3^+^ T cells and %CAR T cells), bacterial and fungal contamination (119). Also, titer, stability, and function of the viral vector should be measured and attended (87). It is noteworthy to attend the name and label of the product before infusion, especially in autologous CAR T cell products.

### Are All Patients Candidate for CAR T Cell Therapy?

Patients with special characteristics would be appropriate for CAR T cell infusion. Some of these conditions include cancer must have a unique target antigen (e.g., CD19), the patient must have appropriate performance condition and enough tolerance to possible side effects. Also, the patient must have an adequate number of T cells to be isolated and engineered. Patients who have experienced severe autoimmune disease do not qualify to participate; this is related to the probability of disease exacerbation during the infusion period time or the immunosuppressive drugs they receive. It is recommended that if a patient’s cancer is in control or remission phase, allogeneic HSCT is preferred than CAR T cell therapy (120).

### Toxicity Control and Follow-Up

Since CAR T cell infusion is accompanied by hazardous toxicities which may endanger patients, hospital admission and observation during infusion period seems to be necessary. In case of B-cell aplasia following CD19 CAR T cell infusion, intravenous immunoglobulin injection must be considered. To prevent and control severe CRS, anti-IL-6, vasopressor support, and hydration strategies are pivotal. If a patient experiences neutropenic fever, supportive care and standard considerations must be performed based on guidelines (120).

Also, to realize the effectiveness of treatment in patients, follow-up is required to measure the rate of stable disease, PR and CR conditions. However, the follow up period has been variable between different studies according to their limitations (116).

## Concluding Remarks

Impressive outcomes have been achieved with CAR T cell therapy especially in patients with CD19^+^ malignancies. This has led to the development of several studies applying CAR T cells in numerous cancers. Although USA and China are responsible for most clinical trials, CAR T cell therapy is growing rapidly all over the world.

Two products including Kymriah by Novartis and Yescarta by Kite Pharma have recently gained FDA approval. Kymriah can be administrated for B-ALL and lymphoma patients and Yescarta for large B-cell lymphoma (121). Improving CAR T cell production protocols, target selection, and clinical considerations may lead to the advent of multiple genetically engineered drugs for different cancers, especially solid tumors.

Results related to clinical trials of different centers are substantially variable which is related to the tumor type, CAR T cell phenotype, production, and application strategies selected. Using the information released by these trials could be beneficial to create new strategies in order to ameliorate targeting, anti-tumor function, tissue penetration, and perseverance of CAR T cells in further studies.

## Future Directions

Adoptive cell therapy using CAR T therapy have been applied for many years but still, some challenges have remained which hinder its optimized function; so, there is a requirement to enhance the efficacy of CAR T cells against tumor cells.

### Cell Type Selection

To improve the existence, proliferation and expansion time of CAR T cells in patients, particular subtypes of less differentiated T cells such as αβ T cells are preferred (101). Moreover, selection of central memory T cells or CD4^+^/CD8^+^ T cells as initiating cell population is considered to decrease the cell variability of the product (46, 87, 122). Blaeschke et al. administrated central and stem memory T cells to engineer 4-1BB CAR T cells against CD19^+^ ALL. This study exhibited that using robust memory composition of T cells for designing CAR T cells can increase the expansion of these cells up to 100-fold. These CAR T cells also showed higher efficacy (123).

NK cells may also be chosen as a target cell to be transduced with the CAR transgene. Chen et al. engineered CD3CAR NK92 cells to target the T cell malignancies which led to the control and suppression of Jurkat tumor cells (124).

### Combination Therapy

It is recently documented that CAR T cell therapy combined with immune checkpoint inhibition could improve its anti-tumor effect (125). Also, CAR T cell therapy accompanied by chemotherapy and HSCT may lead to prolonged survival and a better outcome for patients (126). Altogether, a combination of new treatment strategies with CAR T cell therapy could lead to enhanced anti-tumor efficacy.

### New Target Identification

To enhance the specificity of CAR T cell against tumor cells and reduce the adverse effects, TSAs must be selected. With the identification of new unique targets both for hematological and solid malignancies, improved anti-tumor efficacy and better outcomes are expected.

### From Hematologic Malignancies to Solid Tumors

Application of CAR T cells has represented favorable results with durable immunity especially in hematological malignancies; however, CAR T cell therapy in solid tumors is still in its early stages of the experiment. Multiple trials have aimed to explore the benefit of CAR T cell therapy in solid tumors, but the results have not been impressive. This problem has been attributed to the immunosuppressive microenvironment of the solid tumors that inhibits the activation and function of the CAR T. Specific characteristics of solid tumor microenvironment include obstacles in T cell trafficking, anti-immune function (several immunosuppressive cytokines and immune cells), and fibrotic tissue of solid tumors (presence of fibroblasts). Also, due to the stromal structure of solid tumors, CAR T cells show limited infiltration to the solid tissue (2, 127). A comparison of obstacles of CAR T cell therapy in solid tumors and hematological malignancies and possible solutions have been presented in Table 3.

**Table 3:** A comparison of obstacles and feasible solutions of applying chimeric antigen receptor (CAR) T cells in solid tumors and hematological malignancies.

Challenges in solid tumors	Feasible solutions	Challenges in hematological malignancies	Feasible solutions
Trafficking to the solid tumor site	Local infusion of CAR T cells (29)	Antigen escape	Targeting two antigens (such as CD19/CD20) *via* dual CARs (129)
	Pro-inflammatory chemokine production by CAR T cells (79)		
	Engineering tumor site-specific CAR T cells (e.g., hypoxia-inducible factor sensitized and epidermal growth factor receptor sensitized CARs) (100)		
	Engineering CAR T cells with chemokine receptors (CXCR2, CCR4) (128)		

The immunosuppressive microenvironment of the malignant tumor: cytokines, immune inhibitory checkpoints, and immune cells	Reduction and inhibition of regulatory T cells by lymphodepletion (113)	B-cell aplasia and multiple infections after infusion	Intravenous immunoglobulin injection (60)
	Employment of exogenous interleukin (IL)-2, IL-7, and IL-12 for enhancing CAR T cell efficacy (119)		Dual CD19-Dectin-1 CAR T cells (99)
	Programmed death 1 (PD-1) and cytotoxic T lymphocyte-associated antigen 4 blockade by monoclonal antibody (89, 125)		
	Administering clustered regularly interspaced short palindromic repeats (CRISPR)/CRISPR-associated 9 for engineering PD-1-knockout CAR T cells (68)		
	Designing other inhibitory-molecule knockout CAR T cells		

Target antigen heterogenicity and specificity	Dual CARs targeting two antigens simultaneously (97)	Reduced number of CAR T cells in patient’s blood	Multiple infusions of CAR T product (130)
	Identifying more tumor-specific antigens proprietary for solid tumor		
	Administrating glycan-CARs to increase the tumor-specificity of CAR (78)		
	Indication of specific CARs for patients with particular antigen expression profile		
	Identifying new tumor-unique antigens		

Controlling side effects	Engineering smarter CAR T cells [e.g., inhibitory CARs (iCARs), “on-off switch” CARs, split CARs] (90)	Severe acute side effects	iCARs (90)
	Predicting cytokine release syndrome (CRS) *via* specific biomarkers (e.g., IL6, CRP, and IFNγ) (59)		Predicting CRS *via* specific biomarkers (e.g., IL6, CRP, and IFNγ) (59)
	Administration of anti-IL6 (Tocilizumab) and hydration methods in case of severe toxicity (59)		Administration of anti-IL6 (Tocilizumab) and hydration methods in case of severe toxicity (59)
	Transient expression of CAR (76)		

Limited *in vivo* persistence	Selecting appropriate T cell subgroups		
	Simultaneous infusion of T cell stimulating cytokines (IL-12, IL-15, and IL-18) (59)		

Penetration to the solid tumor stroma	Anti-fibroblast-associated protein-CAR T cells (131)		
	Heparanase expressing CAR T cells (HPSE-CAR) (132)		

### Other Diseases

The extreme potential of CAR T cell therapy has turned it into a promising treatment method against other non-cancerous diseases such as HIV/AIDS, SLE, sepsis, and fungal infections. CAR T cell therapy provides a promising era of ACT for even more diseases.

### Preconditioning Regimen

The preconditioning regimen used for chemotherapy expands the duration of CAR T cells’ function. As a result, some changes in chemotherapy regimen may be useful. Recently, cyclophosphamide has been introduced as a selective chemotherapy regimen. Further researches would determine the best preconditioning cytotoxic agents.

### Modulation of Toxicity

As a powerful cytotoxic treatment method, CAR T cell therapy is followed by some side effects. Although several approaches such as anti-IL-6 (Tocilizumab) have been introduced for CRS diminution, more interventions are required to decrease side effects without disturbing the function of CAR T cells. Also, novel strategies such as employment of suicide genes, dual CARs, transient mRNA transfection, iCARs, and switch CAR T cells can be administrated to terminate the function CAR T cells in case of serious toxicity.

### Autologous or Allogeneic T Cells?

Most studies have utilized autologous T cells to produce CAR T cells; however, some challenges such as higher expense, lower quantity, and quality still exist (67). Although allogeneic CAR T cells have represented impressive outcomes in relapsed hematological malignancies, there is a chance of GVHD after infusion. Accumulating data declare allogeneic CAR T cells to be safe and effective after allogeneic HSCT. Also, GVHD prophylaxis *via* cyclophosphamide has shown to decrease the risk of GVHD (115, 133, 134). Ghosh et al. reported allogeneic CAR T cells to exhibit graft-versus-lymphoma effect accompanied by decreased GVHD (135). Also, administration of allogeneic T cells to produce CAR T cells may vanquish exhaustion and senescence of T cells especially in CLL patients (136).

In conclusion, CAR T cell therapy has been introduced as a novel therapeutic strategy with impressive outcomes, especially in hematological malignancies. Further studies are required to determine whether developing new generations of CAR T cell therapy can shift efforts from cancer treatment to cancer cure!

## Author Contributions

RE, EK, and ST contributed to data gathering, writing the primary draft of the manuscript, and designing figures and tables. AE contributed to the hypothesis, corresponding, scientific and structural editing, and verifying the manuscript before submission.

## Conflict of Interest Statement

The authors declare that the research was conducted in the absence of any commercial or financial relationships that could be construed as a potential conflict of interest.

## References

[1] Johnson SB Park HS Gross CP Yu JB. Use of alternative medicine for cancer and its impact on survival. J Natl Cancer Inst (2018) 110(1):121–4. 10.1093/jnci/djx145 28922780

[2] Figueroa JA Reidy A Mirandola L Trotter K Suvorava N Figueroa A Chimeric antigen receptor engineering: a right step in the evolution of adoptive cellular immunotherapy. Int Rev Immunol (2015) 34(2):154–87. 10.3109/08830185.2015.1018419 25901860

[3] Robbins PF Lu Y-C El-Gamil M Li YF Gross C Gartner J Mining exomic sequencing data to identify mutated antigens recognized by adoptively transferred tumor-reactive T cells. Nat Med (2013) 19(6):747–52. 10.1038/nm.3161 23644516 PMC3757932

[4] Marofi F Vahedi G Biglari A Esmaeilzadeh A Athari SS. Mesenchymal stromal/stem cells: a new era in the cell-based targeted gene therapy of cancer. Front Immunol (2017) 8:1770. 10.3389/fimmu.2017.01770 29326689 PMC5741703

[5] Yu S Li A Liu Q Li T Yuan X Han X Chimeric antigen receptor T cells: a novel therapy for solid tumors. J Hematol Oncol (2017) 10(1):78. 10.1186/s13045-017-0444-9 28356156 PMC5372296

[6] Vasekar M Rizvi S Liu X Vrana KE Zheng H. Novel immunotherapies for hematological malignancies. Curr Mol Pharmacol (2016) 9(3):264–71. 10.2174/1874467208666150716121253 26177640

[7] Gross G Waks T Eshhar Z. Expression of immunoglobulin-T-cell receptor chimeric molecules as functional receptors with antibody-type specificity. Proc Natl Acad Sci U S A (1989) 86(24):10024–8. 10.1073/pnas.86.24.10024 2513569 PMC298636

[8] Dotti G Savoldo B Brenner M Fifteen years of gene therapy based on chimeric antigen receptors: “are we nearly there yet?”. Hum Gene Ther (2009) 20(11):1229–39. 10.1089/hum.2009.142 19702437 PMC2829458

[9] Harris DT Kranz DM. Adoptive T cell therapies: a comparison of T cell receptors and chimeric antigen receptors. Trends Pharmacol Sci (2016) 37(3):220–30. 10.1016/j.tips.2015.11.004 26705086 PMC4764454

[10] Zhang E Xu H. A new insight in chimeric antigen receptor-engineered T cells for cancer immunotherapy. J Hematol Oncol (2017) 10(1):1. 10.1186/s13045-016-0379-6 28049484 PMC5210295

[11] Savoldo B Ramos CA Liu E Mims MP Keating MJ Carrum G CD28 costimulation improves expansion and persistence of chimeric antigen receptor–modified T cells in lymphoma patients. J Clin Invest (2011) 121(5):1822 10.1172/JCI46110 21540550 PMC3083795

[12] Au R. Immunooncology: can the right chimeric antigen receptors T-cell design be made to cure all types of cancers and will it be covered? J Pharm (2017) 2017:7513687. 10.1155/2017/7513687 28239502 PMC5292386

[13] Hoyos V Savoldo B Quintarelli C Mahendravada A Zhang M Vera J Engineering CD19-specific T lymphocytes with interleukin-15 and a suicide gene to enhance their anti-lymphoma/leukemia effects and safety. Leukemia (2010) 24(6):1160–70. 10.1038/leu.2010.75 20428207 PMC2888148

[14] Levine BL Miskin J Wonnacott K Keir C Global manufacturing of CAR T cell therapy. Mol Ther Methods Clin Dev (2017) 4:92–101. 10.1016/j.omtm.2016.12.006 28344995 PMC5363291

[15] Bobis-Wozowicz S Galla M Alzubi J Kuehle J Baum C Schambach A Non-integrating gamma-retroviral vectors as a versatile tool for transient zinc-finger nuclease delivery. Sci Rep (2014) 4:4656. 10.1038/srep04656 24722320 PMC3983605

[16] Maetzig T Galla M Baum C Schambach A. Gammaretroviral vectors: biology, technology and application. Viruses (2011) 3(6):677–713. 10.3390/v3060677 21994751 PMC3185771

[17] Zufferey R Production of Lentiviral Vectors. Heidelberg: Springer (2002). p. 107–21. 10.1007/978-3-642-56114-6_5 11892243

[18] Naldini L Trono D Verma IM Lentiviral vectors, two decades later. Science (2016) 353(6304):1101–2. 10.1126/science.aah6192 27609877

[19] Monjezi R Miskey C Gogishvili T Schleef M Schmeer M Einsele H Enhanced CAR T-cell engineering using non-viral sleeping beauty transposition from minicircle vectors. Leukemia (2017) 31(1):186. 10.1038/leu.2016.180 27491640

[20] Hudecek M Gogishvili T Monjezi R Wegner J Shankar R Kruesemann C Minicircle-Based Engineering of Chimeric Antigen Receptor (CAR) T Cells. Current Strategies in Cancer Gene Therapy. Cham: Springer (2016). p. 37–50. 10.1007/978-3-319-42934-2_3 28101686

[21] Chen Z-Y He C-Y Kay MA. Improved production and purification of minicircle DNA vector free of plasmid bacterial sequences and capable of persistent transgene expression in vivo. Hum Gene Ther (2005) 16(1):126–31. 10.1089/hum.2005.16.126 15703495

[22] Kay MA He C-Y Chen Z-Y. A robust system for production of minicircle DNA vectors. Nat Biotechnol (2010) 28(12):1287. 10.1038/nbt.1708 21102455 PMC4144359

[23] Hung CF Xu X Li L Ma Y Jin Q Viley A Development of anti-human mesothelin-targeted chimeric antigen receptor messenger RNA–transfected peripheral blood lymphocytes for ovarian cancer therapy. Hum Gene Ther (2018) 29(5):614–25. 10.1089/hum.2017.080 29334771 PMC5930796

[24] Zhang C Liu J Zhong JF Zhang X. Engineering CAR-T cells. Biomark Res (2017) 5(1):22. 10.1186/s40364-017-0102-y 28652918 PMC5482931

[25] Manesh ME Esmaeilzadeh A Mirzaei MH IL-24: a novel gene therapy candidate for immune system upregulation in Hodgkin’s lymphoma. J Med Hypoth Ideas (2015) 9(1):61–6. 10.1016/j.jmhi.2014.05.002

[26] Mock U Nickolay L Philip B Cheung GW-K Zhan H Johnston IC Automated manufacturing of chimeric antigen receptor T cells for adoptive immunotherapy using CliniMACS prodigy. Cytotherapy (2016) 18(8):1002–11. 10.1016/j.jcyt.2016.05.009 27378344

[27] Wang X Rivière I. Manufacture of tumor- and virus-specific T lymphocytes for adoptive cell therapies. Cancer Gene Ther (2015) 22(2):85–94. 10.1038/cgt.2014.81 25721207 PMC4480367

[28] Holzinger A Barden M Abken H. The growing world of CAR T cell trials: a systematic review. Cancer Immunol Immunother (2016) 65(12):1433–50. 10.1007/s00262-016-1895-5 27613725 PMC11029082

[29] Adusumilli PS Cherkassky L Villena-Vargas J Colovos C Servais E Plotkin J Regional delivery of mesothelin-targeted CAR T cell therapy generates potent and long-lasting CD4-dependent tumor immunity. Sci Transl Med (2014) 6(261):ra151–261. 10.1126/scitranslmed.3010162 25378643 PMC4373413

[30] Katz SC Burga RA McCormack E Wang LJ Mooring W Point GR Phase I hepatic immunotherapy for metastases study of intra-arterial chimeric antigen receptor–modified T-cell therapy for CEA+ liver metastases. Clin Cancer Res (2015) 21(14):3149–59. 10.1158/1078-0432.CCR-14-1421 25850950 PMC4506253

[31] Slovin SF Wang X Borquez-Ojeda O Stefanski J Olszewska M Taylor C Targeting castration resistant prostate cancer (CRPC) with autologous PSMA-directed CAR+ T cells. Am Soc Clin Oncol (2012) 30:TPS4700 10.1200/jco.2012.30.15\_suppl.tps4700

[32] Li Y-S Wasserman R Hayakawa K Hardy RR. Identification of the earliest B lineage stage in mouse bone marrow. Immunity (1996) 5(6):527–35. 10.1016/S1074-7613(00)80268-X 8986713

[33] Grupp SA Kalos M Barrett D Aplenc R Porter DL Rheingold SR Chimeric antigen receptor–modified T cells for acute lymphoid leukemia. N Engl J Med (2013) 368(16):1509–18. 10.1056/NEJMoa1215134 23527958 PMC4058440

[34] Dai H Zhang W Li X Han Q Guo Y Zhang Y Tolerance and efficacy of autologous or donor-derived T cells expressing CD19 chimeric antigen receptors in adult B-ALL with extramedullary leukemia. Oncoimmunology (2015) 4(11):e1027469. 10.1080/2162402X.2015.1027469 26451310 PMC4590028

[35] Turtle CJ Hanafi L-A Berger C Gooley TA Cherian S Hudecek M CD19 CAR–T cells of defined CD4+: CD8+ composition in adult B cell ALL patients. J Clin Invest (2016) 126(6):2123 10.1172/JCI85309 27111235 PMC4887159

[36] Kochenderfer JN Dudley ME Kassim SH Somerville RP Carpenter RO Stetler-Stevenson M Chemotherapy-refractory diffuse large B-cell lymphoma and indolent B-cell malignancies can be effectively treated with autologous T cells expressing an anti-CD19 chimeric antigen receptor. J Clin Oncol (2014) 33(6):540–9. 10.1200/JCO.2014.56.2025 25154820 PMC4322257

[37] Garfall AL Maus MV Hwang W-T Lacey SF Mahnke YD Melenhorst JJ Chimeric antigen receptor T cells against CD19 for multiple myeloma. N Engl J Med (2015) 373(11):1040–7. 10.1056/NEJMoa1504542 26352815 PMC4646711

[38] Mikkilineni L Kochenderfer JN. Chimeric antigen receptor T-cell therapies for multiple myeloma. Blood (2017) 130(24):2594–602. 10.1182/blood-2017-06-793869 28928126 PMC5731088

[39] Guo B Chen M Han Q Hui F Dai H Zhang W CD138-directed adoptive immunotherapy of chimeric antigen receptor (CAR)-modified T cells for multiple myeloma. J Cell Immunother (2016) 2(1):28–35. 10.1016/j.jocit.2014.11.001

[40] Porter DL Hwang W-T Frey NV Lacey SF Shaw PA Loren AW Chimeric antigen receptor T cells persist and induce sustained remissions in relapsed refractory chronic lymphocytic leukemia. Sci Transl Med (2015) 7(303):ra139–303. 10.1126/scitranslmed.aac5415 26333935 PMC5909068

[41] Wang Q-s Wang Y Lv H-y Han Q-w Fan H Guo B Treatment of CD33-directed chimeric antigen receptor-modified T cells in one patient with relapsed and refractory acute myeloid leukemia. Mol Ther (2015) 23(1):184–91. 10.1038/mt.2014.164 25174587 PMC4426796

[42] Fry TJ Shah NN Orentas RJ Stetler-Stevenson M Yuan CM Ramakrishna S CD22-targeted CAR T cells induce remission in B-ALL that is naive or resistant to CD19-targeted CAR immunotherapy. Nat Med (2018) 24(1):20. 10.1038/nm.4441 29155426 PMC5774642

[43] Zhang W-y Wang Y Guo Y-l Dai H-r Yang Q-m Zhang Y-j Treatment of CD20-directed chimeric antigen receptor-modified T cells in patients with relapsed or refractory B-cell non-Hodgkin lymphoma: an early phase IIa trial report. Signal Transduct Target Ther (2016) 1:16002. 10.1038/sigtrans.2016.2 29263894 PMC5661644

[44] Locke FL Davila ML Regulatory challenges and considerations for the clinical application of CAR-T cell anti-cancer therapy. Expert Opin Biol Ther (2017) 17(6):659–61. 10.1080/14712598.2017.1322953 28454503

[45] Newick K O’Brien S Moon E Albelda SM. CAR T cell therapy for solid tumors. Annu Rev Med (2017) 68:139–52. 10.1146/annurev-med-062315-120245 27860544

[46] Feng K Guo Y Dai H Wang Y Li X Jia H Chimeric antigen receptor-modified T cells for the immunotherapy of patients with EGFR-expressing advanced relapsed/refractory non-small cell lung cancer. Sci China Life Sci (2016) 59(5):468–79. 10.1007/s11427-016-5023-8 26968708

[47] Ahmed N Salsman VS Kew Y Shaffer D Powell S Zhang YJ HER2-specific T cells target primary glioblastoma stem cells and induce regression of autologous experimental tumors. Clin Cancer Res (2010) 16(2):474–85. 10.1158/1078-0432.CCR-09-1322 20068073 PMC3682507

[48] Liu X Zhang N Shi H. Driving better and safer HER2-specific CARs for cancer therapy. Oncotarget (2017) 8(37):62730–41. 10.18632/oncotarget.17528 28977984 PMC5617544

[49] Ahmed N Ratnayake M Savoldo B Perlaky L Dotti G Wels WS Regression of experimental medulloblastoma following transfer of HER2-specific T cells. Cancer Res (2007) 67(12):5957–64. 10.1158/0008-5472.CAN-06-4309 17575166

[50] Ahmed N Salsman VS Yvon E Louis CU Perlaky L Wels WS Immunotherapy for osteosarcoma: genetic modification of T cells overcomes low levels of tumor antigen expression. Mol Ther (2009) 17(10):1779–87. 10.1038/mt.2009.133 19532139 PMC2835000

[51] Sun M Shi H Liu C Liu J Liu X Sun Y. Construction and evaluation of a novel humanized HER2-specific chimeric receptor. Breast Cancer Res (2014) 16(3):R61. 10.1186/bcr3674 24919843 PMC4095682

[52] Ahmed N Brawley VS Hegde M Robertson C Ghazi A Gerken C Human epidermal growth factor receptor 2 (HER2)-specific chimeric antigen receptor-modified T cells for the immunotherapy of HER2-positive sarcoma. J Clin Oncol (2015) 33(15):1688 10.1200/JCO.2014.58.0225 25800760 PMC4429176

[53] Koneru M O’Cearbhaill R Pendharkar S Spriggs DR Brentjens RJ A phase I clinical trial of adoptive T cell therapy using IL-12 secreting MUC-16 ecto directed chimeric antigen receptors for recurrent ovarian cancer. J Transl Med (2015) 13(1):102 10.1186/s12967-015-0460-x 25890361 PMC4438636

[54] Yeku OO Purdon TJ Koneru M Spriggs D Brentjens RJ. Armored CAR T cells enhance antitumor efficacy and overcome the tumor microenvironment. Sci Rep (2017) 7(1):10541. 10.1038/s41598-017-10940-8 28874817 PMC5585170

[55] Neelapu SS Tummala S Kebriaei P Wierda W Gutierrez C Locke FL Chimeric antigen receptor T-cell therapy—assessment and management of toxicities. Nat Rev Clin Oncol (2018) 15(1):47 10.1038/nrclinonc.2018.20 28925994 PMC6733403

[56] Morgan RA Yang JC Kitano M Dudley ME Laurencot CM Rosenberg SA. Case report of a serious adverse event following the administration of T cells transduced with a chimeric antigen receptor recognizing ERBB2. Mol Ther (2010) 18(4):843–51. 10.1038/mt.2010.24 20179677 PMC2862534

[57] Brudno JN Kochenderfer JN. Toxicities of chimeric antigen receptor T cells: recognition and management. Blood (2016) 127(26):3321–30. 10.1182/blood-2016-04-703751 27207799 PMC4929924

[58] Teachey DT Lacey SF Shaw PA Melenhorst JJ Maude SL Frey N Identification of predictive biomarkers for cytokine release syndrome after chimeric antigen receptor T cell therapy for acute lymphoblastic leukemia. Cancer Discov (2016) 6(6):664–79. 10.1158/2159-8290.CD-16-0040 27076371 PMC5448406

[59] Bonifant CL Jackson HJ Brentjens RJ Curran KJ. Toxicity and management in CAR T-cell therapy. Mol Ther Oncolytics (2016) 3:16011. 10.1038/mto.2016.11 27626062 PMC5008265

[60] Davila ML Brentjens RJ. CD19-Targeted CAR T cells as novel cancer immunotherapy for relapsed or refractory B-cell acute lymphoblastic leukemia. Clin Adv Hematol Oncol (2016) 14(10):802–8. 27930631 PMC5536094

[61] Hu Y Sun J Wu Z Yu J Cui Q Pu C Predominant cerebral cytokine release syndrome in CD19-directed chimeric antigen receptor-modified T cell therapy. J Hematol Oncol (2016) 9(1):70. 10.1186/s13045-016-0299-5 27526682 PMC4986179

[62] Czock D Keller F Rasche FM Häussler U. Pharmacokinetics and pharmacodynamics of systemically administered glucocorticoids. Clin Pharmacokinet (2005) 44(1):61–98. 10.2165/00003088-200544010-00003 15634032

[63] Maus MV Haas AR Beatty GL Albelda SM Levine BL Liu X T cells expressing chimeric antigen receptors can cause anaphylaxis in humans. Cancer Immunol Res (2013) 1(1):26–31. 10.1158/2326-6066.CIR-13-0006 PMC3888798 24777247

[64] Scholler J Brady TL Binder-Scholl G Hwang W-T Plesa G Hege KM Decade-long safety and function of retroviral-modified chimeric antigen receptor T cells. Sci Transl Med (2012) 4(132):ra53–53. 10.1126/scitranslmed.3003761 22553251 PMC4368443

[65] Hacein-Bey-Abina S Von Kalle C Schmidt M McCormack M Wulffraat N Leboulch PA LMO2-associated clonal T cell proliferation in two patients after gene therapy for SCID-X1. Science (2003) 302(5644):415–9. 10.1126/science.1088547 14564000

[66] Hacein-Bey-Abina S Garrigue A Wang GP Soulier J Lim A Morillon E Insertional oncogenesis in 4 patients after retrovirus-mediated gene therapy of SCID-X1. J Clin Invest (2008) 118(9):3132. 10.1172/JCI35700 18688285 PMC2496963

[67] Ren J Zhao Y. Advancing chimeric antigen receptor T cell therapy with CRISPR/Cas9. Protein Cell (2017) 8(9):634–43. 10.1007/s13238-017-0410-x 28434148 PMC5563282

[68] Rupp LJ Schumann K Roybal KT Gate RE Chun JY Lim WA CRISPR/Cas9-mediated PD-1 disruption enhances anti-tumor efficacy of human chimeric antigen receptor T cells. Sci Rep (2017) 7(1):737. 10.1038/s41598-017-00462-8 28389661 PMC5428439

[69] Wu Y Jiang S Ying T. From therapeutic antibodies to chimeric antigen receptors (CARs): making better CARs based on antigen-binding domain. Expert Opin Biol Ther (2016) 16(12):1469–78. 10.1080/14712598.2016.1235148 27618260

[70] Wang Z Wu Z Liu Y Han W. New development in CAR-T cell therapy. J Hematol Oncol (2017) 10(1):53. 10.1186/s13045-017-0423-1 28222796 PMC5320663

[71] Rafiq S Purdon T Daniyan A Koneru M Dao T Liu C Optimized T-cell receptor-mimic chimeric antigen receptor T cells directed toward the intracellular Wilms Tumor 1 antigen. Leukemia (2017) 31(8):1788. 10.1038/leu.2016.373 27924074 PMC5495623

[72] Nelson AL. Antibody fragments: hope and hype. MAbs (2010) 2(1):77–83. 10.4161/mabs.2.1.10786 20093855 PMC2828581

[73] Nelson AL Reichert JM Development trends for therapeutic antibody fragments. Nat Biotechnol (2009) 27(4):331–7. 10.1038/nbt0409-331 19352366

[74] Zhang G Wang L Cui H Wang X Zhang G Ma J Anti-melanoma activity of T cells redirected with a TCR-like chimeric antigen receptor. Sci Rep (2014) 4:3571. 10.1038/srep03571 24389689 PMC3880964

[75] Guo Y Feng K Wang Y Han W. Targeting cancer stem cells by using chimeric antigen receptor-modified T cells: a potential and curable approach for cancer treatment. Protein Cell (2018) 9(6):516–26. 10.1007/s13238-017-0394-6 28290053 PMC5966354

[76] Ang WX Li Z Chi Z Du S-H Chen C Tay JC Intraperitoneal immunotherapy with T cells stably and transiently expressing anti-EpCAM CAR in xenograft models of peritoneal carcinomatosis. Oncotarget (2017) 8(8):13545. 10.18632/oncotarget.14592 28088790 PMC5355119

[77] Deng Z Wu Y Ma W Zhang S Zhang Y-Q. Adoptive T-cell therapy of prostate cancer targeting the cancer stem cell antigen EpCAM. BMC Immunol (2015) 16(1):1. 10.1186/s12865-014-0064-x 25636521 PMC4318439

[78] Steentoft C Migliorini D King TR Mandel U June CH Posey AD Jr. Glycan-directed CAR-T cells. Glycobiology (2018) 1:14. 10.1093/glycob/cwy008 29370379

[79] Chmielewski M Abken H. TRUCKs: the fourth generation of CARs. Expert Opin Biol Ther (2015) 15(8):1145–54. 10.1517/14712598.2015.1046430 25985798

[80] Sadelain M Brentjens R Rivière I. The basic principles of chimeric antigen receptor design. Cancer Discov (2013) 3(4):388–98. 10.1158/2159-8290.CD-12-0548 23550147 PMC3667586

[81] Ren J Liu X Fang C Jiang S June CH Zhao Y. Multiplex genome editing to generate universal CAR T cells resistant to PD1 inhibition. Clin Cancer Res (2017) 23(9):2255–66. 10.1158/1078-0432.CCR-16-1300 27815355 PMC5413401

[82] Grigor EJ Fergusson DA Haggar F Kekre N Atkins H Shorr R Efficacy and safety of chimeric antigen receptor T-cell (CAR-T) therapy in patients with haematological and solid malignancies: protocol for a systematic review and meta-analysis. BMJ Open (2017) 7(12):e019321. 10.1136/bmjopen-2017-019321 29288188 PMC5988064

[83] Ahmadi F Esmaeilzadeh A IL-1R2: a novel approach for gene therapy in atherosclerosis. Atherosclerosis (2016) 14(1):e1 10.5779/hypothesis.v14i1.456

[84] Jensen MC Popplewell L Cooper LJ DiGiusto D Kalos M Ostberg JR Antitransgene rejection responses contribute to attenuated persistence of adoptively transferred CD20/CD19-specific chimeric antigen receptor redirected T cells in humans. Biol Blood Marrow Transplant (2010) 16(9):1245–56. 10.1016/j.bbmt.2010.03.014 20304086 PMC3383803

[85] Gargett T Brown MP The inducible caspase-9 suicide gene system as a “safety switch” to limit on-target, off-tumor toxicities of chimeric antigen receptor T cells. Front Pharmacol (2014) 5:235 10.3389/fphar.2014.00235 25389405 PMC4211380

[86] Rodgers DT Mazagova M Hampton EN Cao Y Ramadoss NS Hardy IR Switch-mediated activation and retargeting of CAR-T cells for B-cell malignancies. Proc Natl Acad Sci U S A (2016) 113(4):E459–68. 10.1073/pnas.1524155113 26759369 PMC4743815

[87] Fesnak AD June CH Levine BL. Engineered T cells: the promise and challenges of cancer immunotherapy. Nat Rev Cancer (2016) 16(9):566–81. 10.1038/nrc.2016.97 27550819 PMC5543811

[88] Morsut L Roybal KT Xiong X Gordley RM Coyle SM Thomson M Engineering customized cell sensing and response behaviors using synthetic notch receptors. Cell (2016) 164(4):780–91. 10.1016/j.cell.2016.01.012 26830878 PMC4752866

[89] Scarfò I Maus MV. Current approaches to increase CAR T cell potency in solid tumors: targeting the tumor microenvironment. J Immunother Cancer (2017) 5(1):28. 10.1186/s40425-017-0230-9 28331617 PMC5359946

[90] Fedorov VD Themeli M Sadelain M PD-1- and CTLA-4-based inhibitory chimeric antigen receptors (iCARs) divert off-target immunotherapy responses. Sci Transl Med (2013) 5(215):ra172–215. 10.1126/scitranslmed.3006597 PMC4238416 24337479

[91] Kagoya Y Nakatsugawa M Ochi T Cen Y Guo T Anczurowski M Transient stimulation expands superior antitumor T cells for adoptive therapy. JCI Insight (2017) 2(2):e89580. 10.1172/jci.insight.89580 28138559 PMC5256130

[92] Philip B Kokalaki E Mekkaoui L Thomas S Straathof K Flutter B A highly compact epitope-based marker/suicide gene for easier and safer T-cell therapy. Blood (2014) 124(8):1277–87. 10.1182/blood-2014-01-545020 24970931

[93] Wang X Chang W-C Wong CW Colcher D Sherman M Ostberg JR A transgene-encoded cell surface polypeptide for selection, in vivo tracking, and ablation of engineered cells. Blood (2011) 118(5):1255–63. 10.1182/blood-2011-02-337360 21653320 PMC3152493

[94] Grada Z Hegde M Byrd T Shaffer DR Ghazi A Brawley VS TanCAR: a novel bispecific chimeric antigen receptor for cancer immunotherapy. Mol Ther Nucleic Acids (2013) 2(7):e105. 10.1038/mtna.2013.32 23839099 PMC3731887

[95] Hegde M Grada Z Pignata A Wakefield A Fousek K Bielamowicz K A bispecific chimeric antigen receptor molecule enhances T cell activation through dual immunological synapse formation and offsets antigen escape in glioblastoma. J Immunother Cancer (2015) 3(2):O3 10.1186/2051-1426-3-S2-O3

[96] Lanitis E Poussin M Klattenhoff AW Song D Sandaltzopoulos R June CH Chimeric antigen receptor T Cells with dissociated signaling domains exhibit focused antitumor activity with reduced potential for toxicity in vivo. Cancer Immunol Res (2013) 1(1):43–53. 10.1158/2326-6066.CIR-13-0008 24409448 PMC3881605

[97] Wilkie S van Schalkwyk MC Hobbs S Davies DM van der Stegen SJ Pereira ACP Dual targeting of ErbB2 and MUC1 in breast cancer using chimeric antigen receptors engineered to provide complementary signaling. J Clin Immunol (2012) 32(5):1059–70. 10.1007/s10875-012-9689-9 22526592

[98] Hegde M Mukherjee M Grada Z Pignata A Landi D Navai SA Tandem CAR T cells targeting HER2 and IL13Rα2 mitigate tumor antigen escape. J Clin Invest (2016) 126(8):3036–52. 10.1172/JCI83416 27427982 PMC4966331

[99] Kumaresan PR Albert N Singh H Olivares S Maiti SN Mi T Abstract A193: bioengineered dectin-1 CAR+ T cells to control invasive fungal infection. AACR (2016) 4(1):A193 10.1158/2326-6074.CRICIMTEATIAACR15-A193

[100] Juillerat A Marechal A Filhol JM Valogne Y Valton J Duclert A An oxygen sensitive self-decision making engineered CAR T-cell. Sci Rep (2017) 7:39833. 10.1038/srep39833 28106050 PMC5247770

[101] Chinnasamy D Yu Z Theoret MR Zhao Y Shrimali RK Morgan RA Gene therapy using genetically modified lymphocytes targeting VEGFR-2 inhibits the growth of vascularized syngenic tumors in mice. J Clin Invest (2010) 120(11):3953–68. 10.1172/JCI43490 20978347 PMC2964987

[102] Chinnasamy D Yu Z Kerkar SP Zhang L Morgan RA Restifo NP Local delivery of lnterleukin-12 using T cells targeting VEGF receptor-2 eradicates multiple vascularized tumors in mice. Clinical Cancer Res (2012) 18(6):1672–83. 10.1158/1078-0432.CCR-11-3050 22291136 PMC6390958

[103] Whilding LM Parente-Pereira AC Zabinski T Davies DM Petrovic RM Kao YV Targeting of aberrant αvβ6 integrin expression in solid tumors using chimeric antigen receptor-engineered T cells. Mol Ther (2017) 25(1):259–73. 10.1016/j.ymthe.2016.10.012 28129120 PMC5261028

[104] Ruggeri L Urbani E André P Mancusi A Tosti A Topini F Effects of anti-NKG2A antibody administration on leukemia and normal hematopoietic cells. Haematologica (2016) 101(5):626–33. 10.3324/haematol.2015.135301 26721894 PMC5004363

[105] Chen N Morello A Tano Z Adusumilli PS CAR T-cell intrinsic PD-1 checkpoint blockade: a two-in-one approach for solid tumor immunotherapy. Oncoimmunology (2017) 6(2):e1273302 10.1080/2162402X.2016.1273302 28344886 PMC5353939

[106] Beavis PA Henderson MA Giuffrida L Mills JK Sek K Cross RS Targeting the adenosine 2A receptor enhances chimeric antigen receptor T cell efficacy. J Clin Invest (2017) 127(3):929–41. 10.1172/JCI89455 28165340 PMC5330718

[107] Ligtenberg MA Mougiakakos D Mukhopadhyay M Witt K Lladser A Chmielewski M Coexpressed catalase protects chimeric antigen receptor–redirected T cells as well as bystander cells from oxidative stress-induced loss of antitumor activity. J Immunol (2016) 196(2):759–66. 10.4049/jimmunol.1401710 26673145 PMC4705591

[108] Kakarla S Chow KK Mata M Shaffer DR Song X-T Wu M-F Antitumor effects of chimeric receptor engineered human T cells directed to tumor stroma. Mol Ther (2013) 21(8):1611–20. 10.1038/mt.2013.110 23732988 PMC3734659

[109] Lo A Wang L-CS Scholler J Monslow J Avery D Newick K Tumor-promoting desmoplasia is disrupted by depleting FAP-expressing stromal cells. Cancer Res (2015) 75(14):2800–10. 10.1158/0008-5472.CAN-14-3041 25979873 PMC4506263

[110] Mohammed S Sukumaran S Bajgain P Watanabe N Heslop HE Rooney CM Improving chimeric antigen receptor-modified T cell function by reversing the immunosuppressive tumor microenvironment of pancreatic cancer. Mol Ther (2017) 25(1):249–58. 10.1016/j.ymthe.2016.10.016 28129119 PMC5363304

[111] John LB Devaud C Duong CP Yong CS Beavis PA Haynes NM Anti-PD-1 antibody therapy potently enhances the eradication of established tumors by gene-modified T cells. Clin Cancer Res (2013) 19(20):5636–46. 10.1158/1078-0432.CCR-13-0458 23873688

[112] Teo PY Yang C Whilding LM Parente-Pereira AC Maher J George AJ Ovarian cancer immunotherapy using PD-L1 siRNA targeted delivery from folic acid-functionalized polyethylenimine: strategies to enhance T cell killing. Adv Healthc Mater (2015) 4(8):1180–9. 10.1002/adhm.201500089 25866054

[113] Shank BR Do B Sevin A Chen SE Neelapu SS Horowitz SB. Chimeric antigen receptor T cells in hematologic malignancies. Pharmacotherapy (2017) 37(3):334–45. 10.1002/phar.1900 28079265

[114] Whilding LM Maher J CAR T cell immunotherapy: the path from the by road to the freeway? Mol Oncol (2015) 9(10):1994–2018. 10.1016/j.molonc.2015.10.012 26563646 PMC5528729

[115] Liu J Zhong JF Zhang X Zhang C. Allogeneic CD19-CAR-T cell infusion after allogeneic hematopoietic stem cell transplantation in B cell malignancies. J Hematol Oncol (2017) 10(1):35. 10.1186/s13045-017-0405-3 28143567 PMC5282795

[116] Hartmann J Schüßler-Lenz M Bondanza A Buchholz CJ Clinical development of CAR T cells—challenges and opportunities in translating innovative treatment concepts. EMBO Mol Med (2017) 9:1183–97. 10.15252/emmm.201607485 28765140 PMC5582407

[117] Levine B. Performance-enhancing drugs: design and production of redirected chimeric antigen receptor (CAR) T cells. Cancer Gene Ther (2015) 22(2):79–84. 10.1038/cgt.2015.5 25675873

[118] Sridhar P Petrocca F. Regional delivery of chimeric antigen receptor (CAR) T-cells for cancer therapy. Cancers. (2017) 9(7):92. 10.3390/cancers9070092 28718815 PMC5532628

[119] Wang X Rivière I. Clinical manufacturing of CAR T cells: foundation of a promising therapy. Mol Ther Oncolytics (2016) 3:16015. 10.1038/mto.2016.15 27347557 PMC4909095

[120] Maus MV Levine BL. Chimeric antigen receptor T-cell therapy for the community oncologist. Oncologist (2016) 21(5):608–17. 10.1634/theoncologist.2015-0421 27009942 PMC4861363

[121] DeFrancesco L CAR-T’s forge ahead, despite Juno deaths. Nat Biotechnol (2017) 35:6–7. 10.1038/nbt0117-6b 28072774

[122] Esmaeilzadeh A Ebtekar M Biglari A Mohammad Hassan Z Induction of allogeneic subcutaneous glioma tumor with GL 26 cell line in Balb/c mice. ZUMS J (2012) 20(78):13–22.

[123] Blaeschke F Kaeuferle T Feucht J Weber D Lotfi R Kaiser A Defined central memory and stem memory T cell phenotype of CD4 and CD8 CAR T cells for the treatment of CD19+ acute lymphoblastic leukemia in an automated closed system. Am Soc Hematol (2016) 128:4558.

[124] Chen KH Wada M Firor AE Pinz KG Jares A Liu H Novel anti-CD3 chimeric antigen receptor targeting of aggressive T cell malignancies. Oncotarget (2016) 7(35):56219. 10.18632/oncotarget.11019 27494836 PMC5302909

[125] John LB Kershaw MH Darcy PK. Blockade of PD-1 immunosuppression boosts CAR T-cell therapy. Oncoimmunology (2013) 2(10):e26286. 10.4161/onci.26286 24353912 PMC3862687

[126] Terakura S CAR-T cell therapy in combination with allogeneic stem cell transplantation. J Hematopoietic Cell Transplant (2017) 6(1):1–7. 10.7889/hct.6.1

[127] Lim WA June CH. The principles of engineering immune cells to treat cancer. Cell (2017) 168(4):724–40. 10.1016/j.cell.2017.01.016 28187291 PMC5553442

[128] Perera LP Zhang M Nakagawa M Petrus MN Maeda M Kadin ME Chimeric antigen receptor modified T cells that target chemokine receptor Ccr4 as a therapeutic modality for T-cell malignancies. Am J Hematol (2017) 92(9):892–901. 10.1002/ajh.24794 28543380 PMC5546946

[129] Zah E Lin M-Y Silva-Benedict A Jensen MC Chen YY T cells expressing CD19/CD20 bi-specific chimeric antigen receptors prevent antigen escape by malignant B cells. Cancer Immunol Res (2016) 4(6):498–508. 10.1158/2326-6066.CIR-15-0231 27059623 PMC4933590

[130] Davila ML Riviere I Wang X Bartido S Park J Curran K Efficacy and toxicity management of 19-28z CAR T cell therapy in B cell acute lymphoblastic leukemia. Sci Transl Med (2014) 6(224):ra25–25. 10.1126/scitranslmed.3008226 24553386 PMC4684949

[131] Wang L-CS Lo A Scholler J Sun J Majumdar RS Kapoor V Targeting fibroblast activation protein in tumor stroma with chimeric antigen receptor T cells can inhibit tumor growth and augment host immunity without severe toxicity. Cancer Immunol Res (2014) 2(2):154–66. 10.1158/2326-6066.CIR-13-0027 24778279 PMC4007316

[132] Caruana I Savoldo B Hoyos V Weber G Liu H Kim ES Heparanase promotes tumor infiltration and antitumor activity of CAR-redirected T lymphocytes. Nat Med (2015) 21(5):524. 10.1038/nm.3833 25849134 PMC4425589

[133] Brudno JN Somerville RP Shi V Rose JJ Halverson DC Fowler DH Allogeneic T cells that express an anti-CD19 chimeric antigen receptor induce remissions of B-cell malignancies that progress after allogeneic hematopoietic stem-cell transplantation without causing graft-versus-host disease. J Clin Oncol (2016) 34(10):1112–21. 10.1200/JCO.2015.64.5929 26811520 PMC4872017

[134] Davies JK Singh H Huls H Yuk D Lee DA Kebriaei P Combining CD19 redirection and alloanergization to generate tumor-specific human T cells for allogeneic cell therapy of B-cell malignancies. Cancer Res (2010) 70(10):3915–24. 10.1158/0008-5472.CAN-09-3845 20424114 PMC2873153

[135] Ghosh A Smith M James SE Davila ML Velardi E Argyropoulos KV Donor CD19 CAR T cells exert potent graft-versus-lymphoma activity with diminished graft-versus-host activity. Nat Med (2017) 23(2):242–9. 10.1038/nm.4258 28067900 PMC5528161

[136] Riches JC Davies JK McClanahan F Fatah R Iqbal S Agrawal S T cells from CLL patients exhibit features of T-cell exhaustion but retain capacity for cytokine production. Blood (2013) 121(9):1612–21. 10.1182/blood-2012-09-457531 23247726 PMC3587324

